# Arginine methylation of BRD4 by PRMT2/4 governs transcription and DNA repair

**DOI:** 10.1126/sciadv.add8928

**Published:** 2022-12-07

**Authors:** Liu Liu, Baicheng Lin, Shasha Yin, Lauren E. Ball, Joe R. Delaney, David T. Long, Wenjian Gan

**Affiliations:** ^1^ Department of Biochemistry and Molecular Biology, Medical University of South Carolina, Charleston, SC 29425, USA.; ^2^ Department of Cell and Molecular Pharmacology and Experimental Therapeutics, Medical University of South Carolina, Charleston, SC 29425, USA.

## Abstract

BRD4 functions as an epigenetic reader and plays a crucial role in regulating transcription and genome stability. Dysregulation of BRD4 is frequently observed in various human cancers. However, the molecular details of BRD4 regulation remain largely unknown. Here, we report that PRMT2- and PRMT4-mediated arginine methylation is pivotal for BRD4 functions on transcription, DNA repair, and tumor growth. Specifically, PRMT2/4 interacts with and methylates BRD4 at R179, R181, and R183. This arginine methylation selectively controls a transcriptional program by promoting BRD4 recruitment to acetylated histones/chromatin. Moreover, BRD4 arginine methylation is induced by DNA damage and thereby promotes its binding to chromatin for DNA repair. Deficiency in BRD4 arginine methylation significantly suppresses tumor growth and sensitizes cells to BET inhibitors and DNA damaging agents. Therefore, our findings reveal an arginine methylation–dependent regulatory mechanism of BRD4 and highlight targeting PRMT2/4 for better antitumor effect of BET inhibitors and DNA damaging agents.

## Teaser

PRMT2/4-mediated arginine methylation of BRD4 promotes its chromatin recruitment and biological function.

## INTRODUCTION

Epigenetic signaling, such as histone modifications, plays a crucial role in regulating various biological processes (*1*, *2*). Epigenetic pathways consist of three categorized players: writers that catalyze various modifications on DNA, RNA, and histones; readers that recognize and bind to different modifications through specialized domains; and erasers that remove epigenetic marks (*3*).

Bromodomain-containing protein 4 (BRD4), the most studied member of the bromodomain and extraterminal domain (BET) protein family, is acknowledged as an epigenetic reader. It binds to hyperacetylated chromatin regions through two tandem bromodomains (BD1 and BD2) and subsequently recruits the positive transcription elongation factor b (P-TEFb), mediators, and other transcriptional regulators to facilitate gene expression (*4*–*6*). Growing evidence also reveals that in addition to transcriptional regulation, BRD4 is an important guardian of genome integrity (*7*). Dysregulation of BRD4 has been implicated in a variety of human cancers, including acute myeloid leukemia and breast cancer (*8*, *9*). Therefore, BRD4 is a promising target for anticancer therapeutics. Over the past decade, numerous BET inhibitors (BETi) have been evaluated in clinical trials, but they have achieved limited success as monotherapy due to toxicities and emergence of resistance (*10*–*12*).

Recent studies showed that arginine methylation is another widespread posttranslational modification (PTM), which is as abundant as phosphorylation and ubiquitination and serves as a critical epigenetic mark (*13*, *14*). Protein arginine methyltransferases (PRMTs) function as the writers to catalyze three types of methylation at arginine residue: monomethylarginine (MMA), asymmetric dimethylarginine (ADMA), and symmetric dimethylarginine (SDMA) (*15*). In mammals, nine PRMTs are grouped into three categories according to their catalytic activity. Type I PRMTs, including PRMT1, PRMT2, PRMT3, PRMT4 (also known as CARM1), PRMT6, and PRMT8, generate MMA and further convert it to ADMA. Type II PRMTs, consisting of PRMT5 and PRMT9, register MMA and further catalyze it to SDMA. PRMT7 is the only known type III PRMT that is limited to generating MMA (*16*). These PRMTs promote methylation of histones and nonhistone substrates, through which they regulate diverse cellular processes, including transcription, DNA damage response (DDR), and signal transduction (*17*, *18*). PRMTs have been implicated in cancer progression, and their high expression correlates with poor clinical outcomes in patients with cancer (*19*, *20*). For example, the predominate type I enzyme PRMT1 methylates epidermal growth factor receptor (EGFR) to promote colorectal tumor growth in a xenograft model, and high EGFR methylation is associated with resistance to cetuximab treatment and reduced overall survival in patients with colorectal cancer (*21*). Thus, PRMTs are potential therapeutic targets, and several PRMT5 inhibitors are evaluated in clinical trials (*22*, *23*).

Arginine methylation interplays with other histone modification signals to orchestrate chromatin-dependent processes (*24*). For example, PRMT1-mediated H4R3 methylation facilitates p300-dependent histone acetylation (*25*), while PRMT6-mediated H3R2 methylation blocks the binding of H3K4me3 readers (*26*). It remains unknown whether there is cross-talk between arginine methylation and BRD4. Here, we demonstrate that PRMT2/4-mediated arginine methylation of BRD4 represents a critical step for its binding of acetylated histones/chromatin to control transcription and DNA repair, which, in turn, promotes BRD4 oncogenic function and decreases sensitivity to BETi and DNA damaging agents.

## RESULTS

### PRMT2 and PRMT4 catalyze arginine methylation of BRD4

**Fig. 1.**
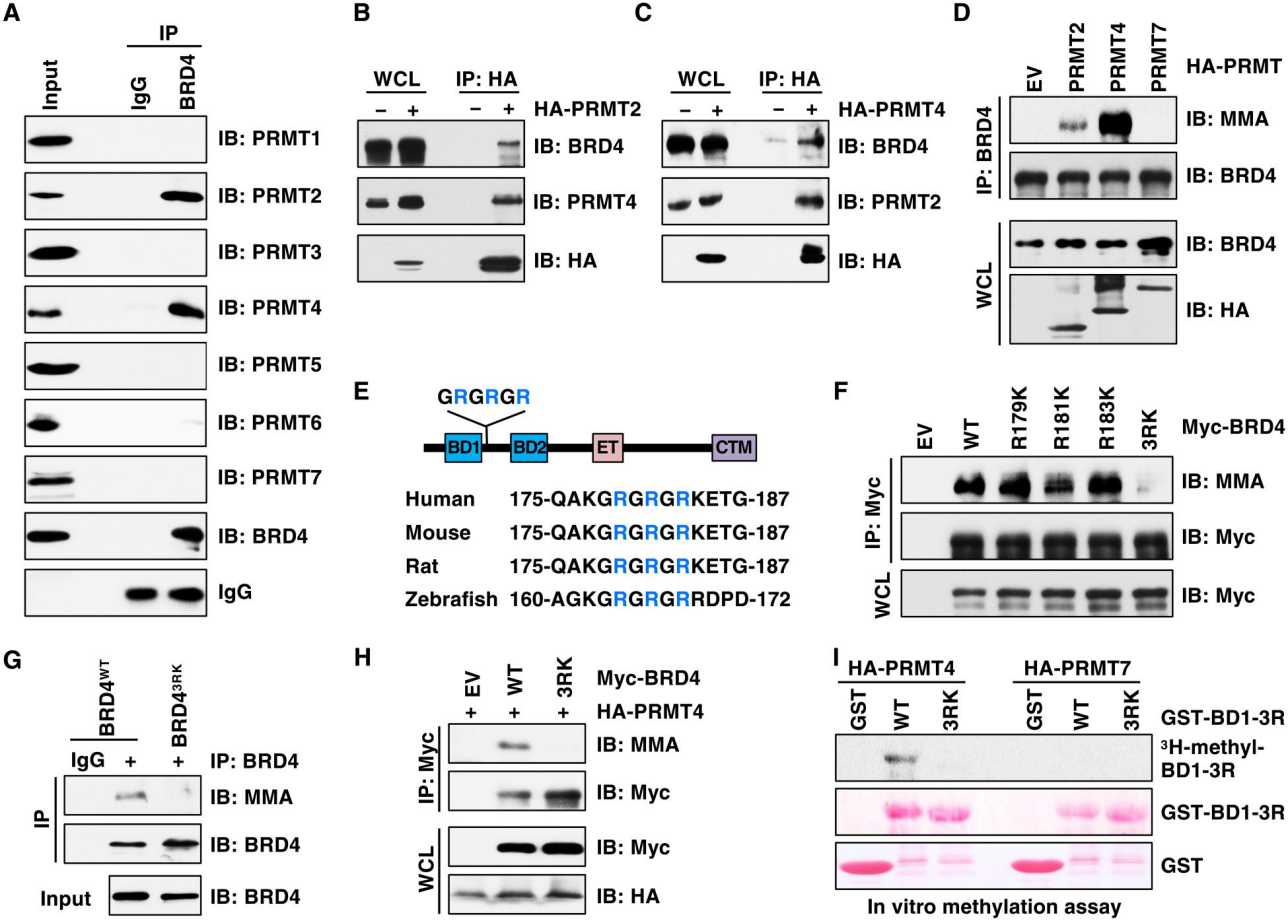
PRMT2 and PRMT4 interact and methylate BRD4. (**A**) Immunoblot (IB) analysis of whole-cell lysates (WCL; input) and BRD4 immunoprecipitation (IP) products derived from MCF7 cells. Immunoglobulin G (IgG) was used as a negative control. (**B**) IB analysis of WCL and hemagglutinin (HA)–tag IP products derived from HEK293T cells transfected with or without HA-PRMT2. (**C**) IB analysis of WCL and HA-tag IP products derived from HEK293T cells transfected with or without HA-PRMT4. (**D**) IB analysis of WCL and BRD4 IP products derived from HEK293T cells transfected with HA-PRMT2, PRMT4, and PRMT7. EV, empty vector. (**E**) A schematic presentation of BRD4 domains and three arginine residues (3R) of BRD4 in various species. (**F**) IB analysis of WCL and Myc tag IP products derived from HEK293T cells transfected with Myc-BRD4-WT, R179K, R181K, R183K, and 3RK. (**G**) IB analysis of WCL and BRD4 IP products derived from BRD4^WT^ and BRD4^3RK^ knock-in MDA-MB-231 cells. IgG was used as a negative control. (**H**) IB analysis of WCL and Myc tag IP products derived from HEK293T cells transfected with HA-PRMT4 and Myc-BRD4-WT or Myc-BRD4-3RK. (**I**) In vitro arginine methylation assays using recombinant GST-BD1-3R and GST-BD1-3RK protein as substrates. HA-PRMT4 and HA-PRMT7 were immunopurified from HEK293T cells and used as methyltransferases.

To determine a possible link between arginine methylation and BRD4, we first investigated the interaction between BRD4 and PRMTs. Western blot using validated antibodies (*27*) showed that both endogenous BRD4 and ectopically expressed BRD4 coimmunoprecipitated PRMT2 and PRMT4 (PRMT2/4 hereafter) but not the other PRMTs (Fig. 1A and fig. S1A). Reciprocally, PRMT2 coimmunoprecipitated BRD4 and PRMT4 (Fig. 1B and fig. S1B), while PRMT4 coimmunoprecipitated BRD4 and PRMT2 (Fig. 1C and fig. S1C). These results demonstrate that PRMT2, PRMT4, and BRD4 form a ternary complex. Given that different domains of BRD4 and PRMT2/4 have distinct functions, we further identified the domain(s) responsible for their interactions. PRMT2 contains an N-terminal Src homology 3 (SH3) domain that recognizes proline-rich motif (PRM) in its partners (*28*, *29*). We found that deletion of the SH3 domain impaired PRMT2 interaction with BRD4 (fig. S1D). PRMT4 contains an N-terminal Pleckstrin homology (PH) domain that regulates protein-protein associations, a catalytic core (CC) composed of a Rossmann fold and a β barrel domain that binds S-adenosylmethionine (SAM), substrate, and a C-terminal region (*30*). Both the PH domain and CC, but not the C-terminal region, interacted with BRD4 (fig. S1E). BRD4 comprises multiple domains: two tandem bromodomains (BD1 and BD2) at the N terminus, one extra terminal (ET) domain that recruits transcriptional effectors including JMJD6 and NSD3, a PRM domain, and a C-terminal motif that interacts with P-TEFb (fig. S1F) (*31*). We showed that the fragment F4 (731 to 1046 amino acids) containing PRM domain is necessary and sufficient for BRD4 interaction with PRMT2 (fig. S1, G and H), which is consistent with the SH3 domain of PRMT2 as a PRM reader. Differently, the fragment F3 (471 to 730 amino acids) containing ET domain is responsible for the interaction between BRD4 and PRMT4 (fig. S1, I and J). These data demonstrate that PRMT2 and PRMT4 form complex with BRD4 through different domains (fig. S1K). In addition to the full length of BRD4 (generally known as long isoform, BRD4-L), there is a short isoform of BRD4 (BRD4-S) that contains only the 1 to 719 amino acids of BRD4-L and unique three C-terminal residues (Glycine, Proline, and Alanine) (fig. S1K) (*6*). Because the BRD4-S contains the ET domain but lacks the PRM domain, it only interacted with PRMT4 but not with PRMT2 (fig. S1, L and M).

To test whether BRD4 is methylated by PRMT2/4, we performed Western blot using recently developed antibodies against MMA or ADMA (*32*). Overexpression of PRMT4 and, to a lesser extent, PRMT2, but not PRMT7, promoted MMA formation on BRD4 in an enzymatic-dependent manner (Fig. 1D and fig. S2, A to E). Compared to expression of individual PRMTs, coexpression of PRMT2 and PRMT4 increased BRD4 MMA signal (fig. S2F). Notably, the ADMA signal was not detected using three widely used pan anti-ADMA antibodies, including anti-ADMA (*32*), adme-R (Cell Signaling Technology), and ASYM24 (Sigma-Aldrich). However, we could not rule out ADMA modification on BRD4 because it might not be recognized by these pan anti-ADMA antibodies. Thus, we focused on MMA of BRD4 in this study. We found that the BRD4 MMA signal was decreased in PRMT2- or PRMT4-depleted cells but was almost abolished in cells depleted of both (fig. S2G). Consistently, the MMA levels of BRD4 were markedly reduced by two selective PRMT4 inhibitors (*33*, *34*), TP-064 (fig. S2, H and I) and EZM2302 (fig. S2, J and K), at doses that efficiently decrease the methylation of a well-characterized PRMT4 substrate, PABP1 (me-PABP1) (*35*). These results demonstrate that both PRMT2 and PRMT4 contribute to BRD4 MMA formation.

Previous proteomic mass spectrometric analysis identified several methylation sites on BRD4, including R179, R181, and R183 (termed 3R hereafter), in human embryonic kidney (HEK) 293 cells and peripheral blood lymphocyte–derived T cells, whereas the PRMTs for these sites remain to be defined (*14*, *36*). Protein sequence alignment showed that 3R are highly conserved in various species (Fig. 1E) but are not present in other BET proteins (BRD2, BRD3, and BRDT). Notably, the 3R are not typical PRMT4 methylation sites that are commonly within a PGM-rich (proline, glycine, and methionine) motif (*37*). We speculate that PRMT2 or other factors may be involved in making BRD4 as a special PRMT4 substrate. To identify whether 3R are the major methylation sites of BRD4, we generate arginine to lysine (RK) mutations and found that substitution of 3R by K (3RK), but not the replacement of individual R by K, completely abolished BRD4 MMA formation in cells (Fig. 1F). Moreover, BRD4-S was also modified by MMA, which was abrogated by 3RK mutations (fig. S3A). We further confirmed monomethylation of R179 by liquid chromatography–tandem mass spectrometry (LC-MS/MS) (fig. S3B), although methylation of R181 and R183 was not detected due to technical issues (See the “Mass spectrometric analysis of BRD4-3R methylation” section). To determine whether 3R methylation occurs at an endogenous level, we used the CRISPR-Cas9 genome editing to introduce 3RK mutations into the endogenous *BRD4* locus (termed BRD4^3RK^) and validated by DNA sequencing (fig. S3C). Notably, compared to BRD4^WT^ cells, the MMA of BRD4 was undetectable in BRD4^3RK^ cells (Fig. 1G), which phenocopied the loss of PRMT2/4 (fig. S2G).

Next, we found that the 3RK mutant abolished BRD4 MMA formation by PRMT2/4 in cells (Fig. 1H and fig. S3D). In vitro methylation assays showed that PRMT4, but not PRMT2 nor PRMT7, catalyzed BRD4 methylation in a 3R-dependent manner (Fig. 1I and fig. S3, E and F). PRMT2 has low/no methyltransferase activity compared to other type I PRMTs (*38*–*40*), and it interacts with PRMT1 to promote PRMT1 activity (*41*). It is possible that PRMT2 serves as a weak methyltransferase or coactivator of PRMT4 to catalyze BRD4 MMA formation. In keeping with the findings in cells (fig. S2, F and G), addition of PRMT2 enhanced PRMT4-mediated BRD4 methylation in vitro (fig. S3G), and in contrast, depletion of PRMT2 decreased PRMT4 activity on methylating BRD4 (fig. S3, H and I). Collectively, these results suggest that PRMT2/4 forms a complex to optimally catalyze MMA of BRD4 at 3R.

### PRMT2/4 and BRD4 coregulate a subset of genes

**Fig. 2.**
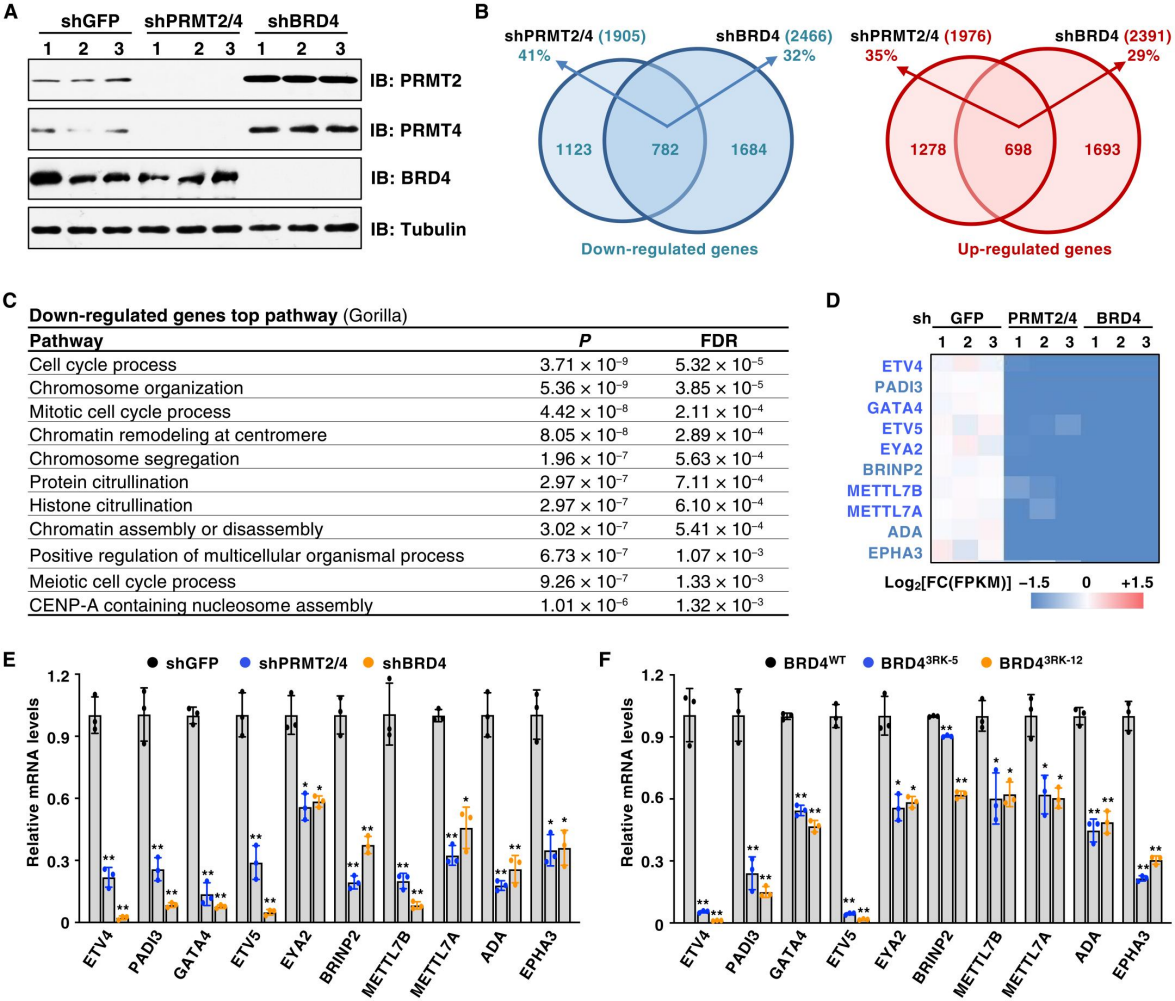
PRMT2/4-mediated BRD4 methylation regulates selective transcriptional program. (**A**) IB analysis of WCL derived from MCF7 cells depleted of PRMT2/4 or BRD4 by shRNA. shGFP as a negative control. (**B**) Venn diagrams of genes within the top and bottom quintiles of shPRMT2/4 and shBRD4 with significant adjusted *P* < 0.05. (**C**) Identification of top pathways down-regulated by BRD4 and PRMT2/4. FDR, false discovery rate; CENP-A, centromere protein A. (**D**) The 10 most down-regulated common genes (mean of shPRMT2/4 and shBRD4) are shown in a heatmap for all samples. (**E**) qRT-PCR analysis of mRNA levels of the down-regulated common genes in MCF7 cells depleted of PRMT2/4 or BRD4. mRNA levels of these genes were normalized to glyceraldehyde-3-phosphate dehydrogenase (GAPDH) and compared to those of shGFP. Data are means ± SD (*n* = 3). *P < 0.05 and **P < 0.01, two-tailed *t* test. (**F**) qRT-PCR analysis of RNA levels of the down-regulated common genes in BRD4^WT^ and BRD4^3RK^ knock-in MDA-MB-231 cells. mRNA levels of these genes were normalized to GAPDH and compared to those of BRD4^WT^. Data are means ± SD (*n* = 3). *P < 0.05 and **P < 0.01, two-tailed *t* test.

Both BRD4 and PRMT2/4 are known as general transcriptional coactivators and play a major role in transcription regulation (*6*, *16*). Having demonstrated that BRD4 is a downstream substrate of PRMT2/4, we next investigated the transcription program in cells depleted of PRMT2/4 or BRD4 (Fig. 2A). RNA sequencing (RNA-seq) analysis revealed a similar gene expression pattern in cells depleted of BRD4 or PRMT2/4 (fig. S4, A and B), suggesting that they may commonly control a subset of gene transcription. PRMT2/4 and BRD4 shared 782 down-regulated genes and 698 up-regulated genes, which accounted for 41 and 32% or 35 and 29% of genes regulated by PRMT2/4 and BRD4, respectively (Fig. 2B). Gene Ontology (GO) enrichment analysis showed that cell cycle, chromosome organization, and chromatin remodeling are among the top suppressed pathways (Fig. 2C), which is consistent with the important functions of BRD4 on cell cycle control and chromatin remodeling (*7*). Notably, 6 of the 10 most down-regulated genes were directly involved in transcriptional regulation (Fig. 2D), including transcription factors [ETS tranlocation variant 4 (ETV4), ETS tranlocation variant 5 (ETV5), and GATA binding protein 4 (GATA4)], coactivator [EYA transcription coactivator and phosphatase 2 (EYA2)], and putative RNA methyltransferases [methyltransferase like 7A (METTL7A) and methyltransferase like 7B (METTL7B)]. We validated these top 10 down-regulated genes in cells depleted of PRMT2/4 or BRD4 by reverse transcription quantitative real-time polymerase chain reaction (qRT-PCR) (Fig. 2E). Codepletion of PRMT2/4 had an additional effect on the expression of some, but not all these genes, compared to depletion of PRMT2 or PRMT4 alone (fig. S4C), indicating that PRMT2 and PRMT4 coregulate a subset of targets while also display their specificity on transcriptional regulation. Moreover, using the doses that notably decrease me-PABP1 (fig. S4D), we found that TP-064 also significantly decreases the expression of these genes (fig. S4, E and F). These 10 genes, particularly ETV4 and ETV5, were significantly decreased in BRD4^3RK^ cells compared to BRD4^WT^ cells (Fig. 2F). Together, these results suggest that PRMT2/4-mediated methylation of BRD4 at 3R plays a critical role in BRD4-mediated transcriptional regulation.

### PRMT2/4-mediated arginine methylation promotes the binding of BRD4 to chromatin

**Fig. 3.**
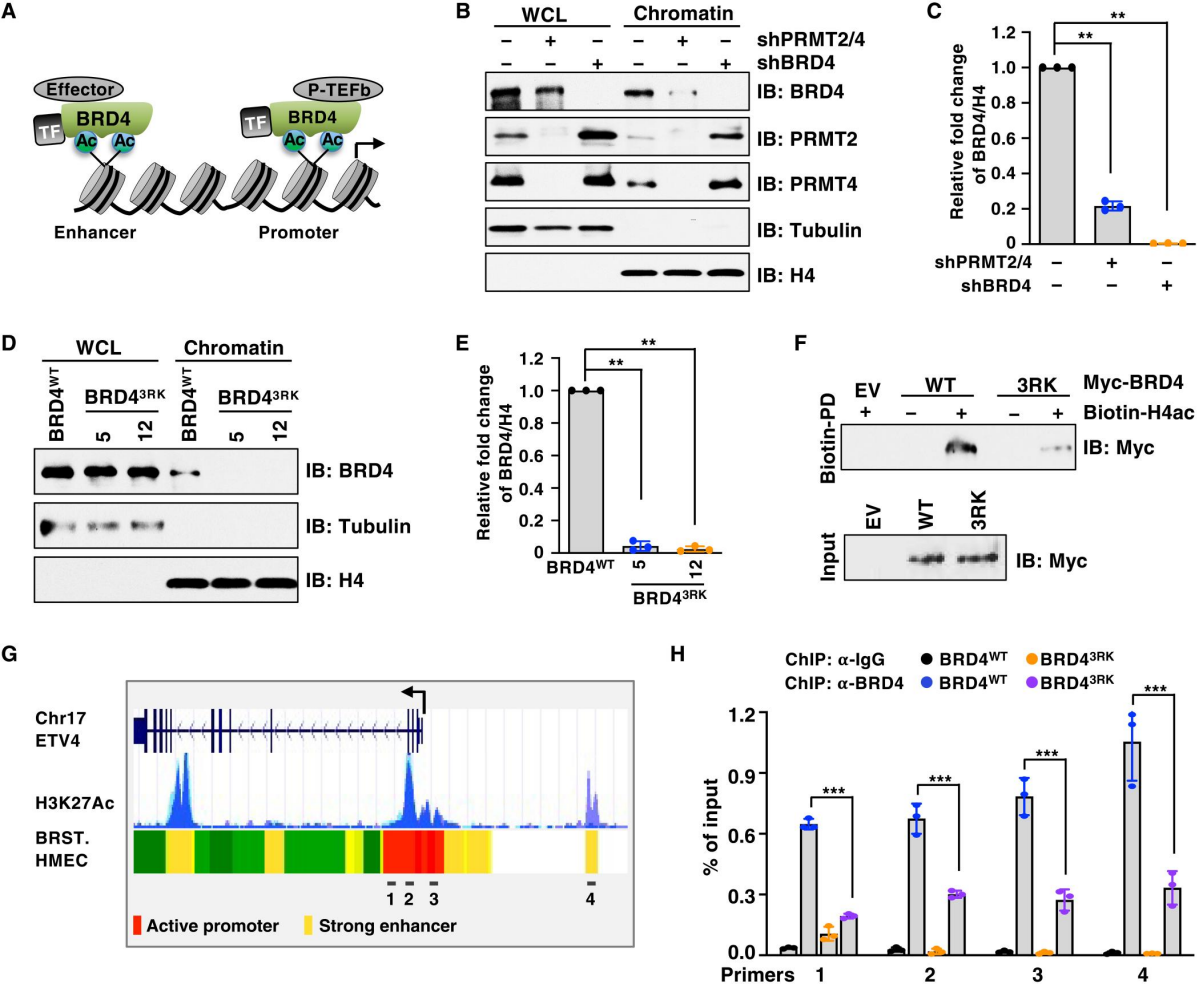
BRD4 arginine methylation is required for its binding to acetylated histone and enrichment on enhancer and promoter. (**A**) A schematic model of BRD4 interaction with acetylated histone and recruitment of transcritional regulators to enhancer and promoter. TF, transcription factors. (**B**) IB analysis of WCL and chromatin fraction derived from MDA-MB-231 cells depleted of PRMT2/4 or BRD4. (**C**) Quantification of chromatin-bound BRD4 in (B), which was normalized to H4 and compared to those of control (without depletion of PRMT2/4 and BRD4). Data are means ± SD (*n* = 3). **P < 0.01, two-tailed *t* test. (**D**) IB analysis of WCL and chromatin fraction derived from BRD4^WT^ and BRD4^3RK^ knock-in MDA-MB-231cells. (**E**) Quantification of chromatin-bound BRD4 in (D), which was normalized to H4 and compared to those of BRD4^WT^. Data are means ± SD (*n* = 3). **P < 0.01, two-tailed *t* test. (**F**) IB analysis of Biotin pulldown (PD) products by acetylated histone H4 peptide. The nuclear fractions derived from HEK293T cells transfected with indicated constructs were used for pulldown assays. (**G**) A schematic presentation of ETV4 gene with a H3K27Ac ChIP-seq track and its associated enhancers/promoters identified by ChromHMM in human mammary epithelial cells (HMECs). Primer pairs targeted promoter (1 to 3) and enhancer (4) for qPCR are indicated. BRST., breast. (**H**) ChIP-qPCR analysis of BRD4 binding to the promoter and enhancer of ETV4 in BRD4^WT^ and BRD4^3RK^ knock-in MDA-MB-231 cells. Data are means ± SD (*n* = 3). ***P < 0.001, two-tailed *t* test.

As an acetyl-lysine reader via the BD domains, BRD4 is enriched on hyperacetylated chromatin regions including enhancers and promoters, thereby serves as a platform for recruitment of various transcriptional regulators (Fig. 3A) (*6*). We next investigated whether PRMT2/4-mediated 3R methylation regulates BRD4 recognition of acetylated histones/chromatin. To this end, we found that chromatin-associated BRD4 was markedly reduced in PRMT2/4-depleted cells (Fig. 3, B and C, and fig. S5A). Consistently, treating cells with TP-064 (fig. S5B) and EZM2302 (fig. S5, C and D) diminished the interaction between BRD4 and chromatin, which phenocopied the effects of BET inhibition (fig. S5E). The BRD4-3RK mutant largely lost the capacity to associate with chromatin (Fig. 3, D and E). Notably, the acetylation of histones H3 and H4 was not notably affected in PRMT2/4-depleted cells or BRD4^3RK^ cells (fig. S5, F and G), supporting the notion that the reduced binding of BRD4 to chromatin in these cells was not due to general defects in histone acetylation. Moreover, compared to BRD4 wild type (WT), individual RK mutants and the 3RK mutant displayed similar capacity of interaction with its partners, including P-TEFb (CDK9 and Cyclin T1) and NSD3 (fig. S5H), suggesting that BRD4-3RK mutations unlikely lead to a global conformational change of BRD4. Next, we examined the interaction between BRD4 and a synthetic histone H4 peptide (H4ac) that is acetylated at K5, K8, K12, and K16 residues by peptide pull-down assay. Only BRD4-WT, but not the BRD4-3RK mutant, bound to H4ac (Fig. 3F and fig. S5I). These results suggest that methylation of BRD4 at 3R is required for its recruitment to acetylated histones/chromatin.

To further understand how PRMT2/4-mediated 3R methylation regulates the transcriptional function of BRD4, we performed chromatin immunoprecipitation quantitative real-time PCR (ChIP-qPCR) to analyze BRD4 recruitment to the enhancer and promoter regions of ETV4, which is a common downstream target of BRD4 and PRMT2/4 (Fig. 2D). Notably, BRD4 occupancy at the enhancer and promoter region of ETV4 was significantly reduced in BRD4^3RK^ cells compared to BRD4^WT^ cells (Fig. 3, G and H). These results suggest that PRMT2/4-mediated methylation facilitates BRD4 enrichment on enhancers and promoters to promote transcription.

### Arginine methylation of BRD4 is required for DNA repair

Emerging evidence demonstrates that BRD4 also functions as a critical keeper of genome integrity. It regulates DDR through both transcriptional-dependent and transcriptional-independent mechanisms (*7*). Consistent with previous studies (*42*, *43*), GO term enrichment analysis of RNA-seq data derived from cells depleted of BRD4 or PRMT2/4 showed that several DNA repair–related pathways were enriched (false discovery rate ≤ 0.05) for pathway suppression (fig. S6A). Twenty-eight DNA repair genes were commonly down-regulated upon knockdown of BRD4 or PRMT2/4 (fig. S6B). These data suggest that PRMT2/4 is involved in BRD4-mediated transcriptional regulation of DNA repair.

**Fig. 4.**
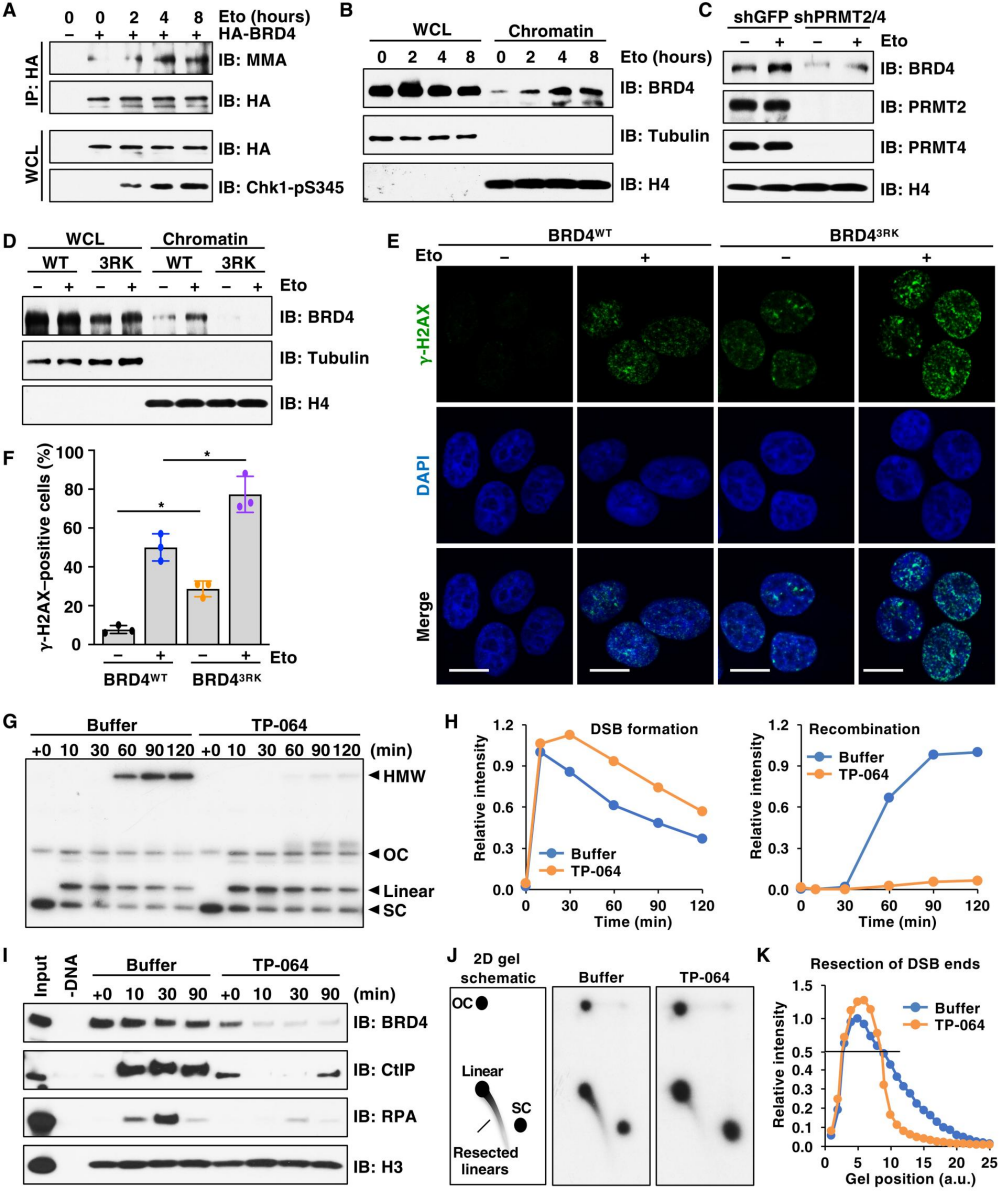
BRD4 arginine methylation is critical for the DDR. (**A**) IB analysis of WCL and IP products derived from MCF7 cells stably expressing HA-BRD4. Cells were treated with 10 μM etoposide (Eto) for 0 to 8 hours before harvesting. (**B**) IB analysis of WCL and chromatin derived from MCF7 cells treated with Eto for 0 to 8 hours before harvesting. (**C**) IB analysis of chromatin derived from MCF7 cells depleted of PRMT2/4 or control (shGFP). Cells were treated with 10 μM Eto for 2 hours before harvesting. (**D**) IB analysis of WCL and chromatin derived from BRD4^WT^ and BRD4^3RK^ knock-in cells treated with 10 μM Eto for 2 hours before harvesting. (**E**) Immunofluorescent images of γ-H2AX in BRD4^WT^ and BRD4^3RK^ knock-in cells treated with 10 μM Eto for 60 min. Scale bars, 10 μm. DAPI, 4′,6-diamidino-2-phenylindole. (**F**) Quantification of γ-H2AX–positive cells from (E). *P < 0.05, two-tailed *t* test. (**G**) 1D agarose gel electrophoresis analysis of DNA intermediates. pDSB was replicated in extract containing [α-^32^P]dATP and supplemented with buffer or 100 μM TP-064 for 45 min before adding Age I to induce DSBs (*T* = +0 min). HMW, high–molecular weight intermediates formed by HR; OC, open circular plasmid; SC, super coiled plasmid. (**H**) Quantitation of linear and HMW molecules from (G). (**I**) IB analysis of input and DNA-bound proteins isolated from extract. (**J**) 2D agarose gel electrophoresis of DNA intermediates. Samples were prepared as (G). (**K**) Quantitation of linear and resected molecules in (J). a.u., arbitrary units.

To explore the relationship between PRMT2/4-mediated BRD4 methylation and DDR, we induced DNA damage using a topoisomerase II inhibitor, etoposide, which has been used in PRMT1/5-relevant studies (*44*, *45*) and is widely used for cancer treatment (*46*). We observed an induction of BRD4 MMA in response to etoposide treatment (Fig. 4A), suggesting that DNA damage signal is an upstream input of BRD4 arginine methylation. Moreover, etoposide treatment increased BRD4 recruitment to chromatin (Fig. 4B), which was reversed by depletion of PRMT2/4 or treatment of TP-064 (Fig. 4C and fig. S6C). Furthermore, compared to BRD4-WT, the BRD4-3RK mutant was almost incapable of loading on chromatin even in the presence of etoposide (Fig. 4D). Given that BRD4 serves as a chromatin platform for recruitment and stabilization of DNA repair complexes (*43*, *47*), these data argue that DNA damage–induced BRD4-3R methylation promotes BRD4 association with chromatin and subsequently facilitates the assembly of DDR complexes to repair damaged DNA. In an agreement with this idea, etoposide-induced γ-H2AX foci were significantly increased in BRD4^3RK^ cells or TP-064–treated cells (Fig. 4, E and F, and fig. S6D). We also observed an elevation of γ-H2AX foci in these cells even without etoposide treatment (Fig. 4, E and F, and fig. S6D), suggesting that inhibition of BRD4 3R methylation and binding to chromatin induce intrinsic DNA damage. These results are consistent with a previous study showing that dissociation of BRD4 from chromatin by JQ1 led to increased DNA damage (*42*).

We then used *Xenopus* egg extract system (*48*) to further explore the role of PRMT2/4-mediated BRD4 methylation in DNA repair. We first confirmed the MMA formation of endogenous BRD4 in the extract (fig. S6E), which was reduced by treatment of TP-064 (fig. S6F). Moreover, BRD4 MMA was increased in response to double-strand breaks (DSBs) induced by Age I cleavage of a plasmid substrate incubated in extracts (fig. S6G). These data phenocopied the observation in cells, demonstrating that *Xenopus* egg extract system is a reliable model to study methylation and DNA damage.

Notably, supplementation of TP-064 in extracts delayed the resolution of DSBs and abolished the accumulation of high–molecular weight (HMW) intermediates that are formed by homologous recombination (HR) (Fig. 4, G and H) (*48*). In reflecting a failure of DNA repair, phosphorylation of the damage response protein Chk1 was enhanced by TP-064 treatment (fig. S6H). Mechanically, TP-064 reduced the loading of BRD4, as well as the resection nuclease CtBP interacting protein (CtIP) and the single-stranded DNA binding protein replication protein A (RPA) to DNA (Fig. 4I), suggesting that DNA end resection was defective. We then analyzed DNA ends directly using two-dimensional (2D) agarose gel electrophoresis, which showed that resection was severely reduced by TP-064 treatment (Fig. 4, J and K). These results are consistent with our recent finding that BRD4 promotes resection of DNA ends to facilitate HR (*49*) and also suggest that PRMT4 facilitates DSB end processing and HR in part by promoting BRD4 methylation and its association with chromatin.

### Deficiency in BRD4-3R methylation attenuates its oncogenic function

**Fig. 5.**
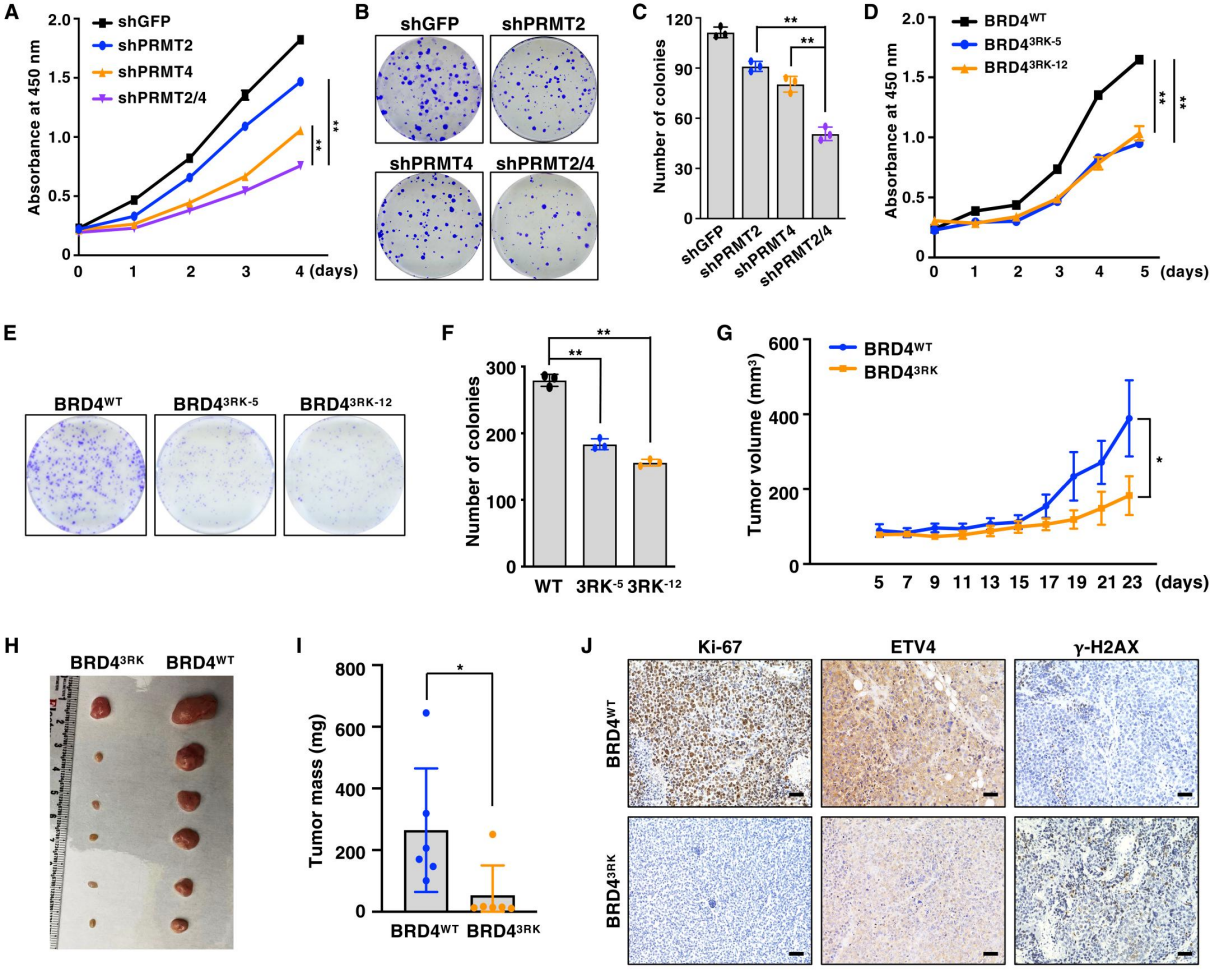
Deficiency in BRD4 arginine methylation attenuates cell proliferation and tumor growth. (**A**) MDA-MB-231 cells depleted of PRMT2, PRMT4, or both (shGFP as a negative control) were subjected to cell proliferation assays. Data are means ± SD (*n* = 3). **P < 0.01, two-way analysis of variance (ANOVA). (**B** and **C**) MDA-MB-231 cells depleted of PRMT2, PRMT4, or both (shGFP as a negative control) were subjected to colony formation assays. Representative images were shown in (B). Colonies were quantified in (C). Data are means ± SD (*n* = 3). **P < 0.01, two-tailed *t* test. (**D**) BRD4^WT^ and BRD4^3RK^ knock-in MDA-MB-231 cells were subjected to cell proliferation assays. Data are means ± SD (*n* = 3). **P < 0.01, two-way ANOVA. (**E** and **F**) BRD4^WT^ and BRD4^3RK^ knock-in MDA-MB-231 cells were subjected to colony formation assays. Representative images were shown in (E). Colonies were quantified in (F). Data are means ± SD (*n* = 3). **P < 0.01, two-tailed *t* test. (**G**) BRD4^WT^ and BRD4^3RK^ knock-in MDA-MB-231 cells were subjected to mouse xenograft assays. Tumor size was measured every other day for 23 days. Data are means ± SEM of *n* = 6. *P < 0.05, two-way ANOVA. (**H** and **I**) Dissected tumors were weighed. Data are means ± SD of *n* = 6. *P < 0.05, two-tailed *t* test. (**J**) Immunohistochemistry (IHC) staining for Ki-67, ETV4, and γ-H2AX in xenograft tumors. Scale bars, 50 μm.

Given that PRMT2/4 and BRD4 are commonly overexpressed in breast tumors and play a crucial role in breast cancer progression (*50*–*52*), we next investigated whether PRMT2/4-mediated methylation of BRD4 at 3R regulates its oncogenic function using two common breast cancer cell lines, MCF7 and MDA-MB-231, that have been frequently used in PRMT4- and BRD4-relevant studies (*52*–*55*). Short hairpin RNA (shRNA)–mediated knockdown of PRMT2 or PRMT4 alone inhibits cell proliferation and colony formation, while codepletion of PRMT2/4 has an additive effect (Fig. 5, A to C). We also confirmed that simultaneous depletion of PRMT2/4 by CRISPR-Cas9 significantly suppresses cell proliferation and colony formation (fig. S7, A to F). Notably, compared to BRD4^WT^ cells, BRD4^3RK^ cells displayed a significant reduction in cell proliferation (Fig. 5D) and colony formation (Fig. 5, E and F). To further explore the in vivo role of BRD4-3R methylation, the BRD4^WT^ and BRD4^3RK^ cells were injected into immunodeficient nude mice. We found that both tumor growth rate and tumor mass were much lower in the BRD4^3RK^ group than in the BRD4^WT^ group (Fig. 5, G to I). Consistent with the role of BRD4-3R methylation in regulating ETV4 expression (Fig. 2F) and intrinsic DNA damage (Fig. 4E), immunohistochemistry analysis showed that BRD4^3RK^ tumors exhibited lower expression of ETV4 and the proliferation marker Ki-67 but higher γ-H2AX signal than BRD4^WT^ tumors (Fig. 5J). Together, these results demonstrate that PRMT2/4-mediated methylation of BRD4 promotes cell proliferation and tumorigenesis, in part, by activating the oncogenic transcription program and preventing accumulation of DNA damage.

### PRMT2/4-mediated methylation of BRD4 dictates sensitivity to BETi and DNA damaging agents

**Fig. 6.**
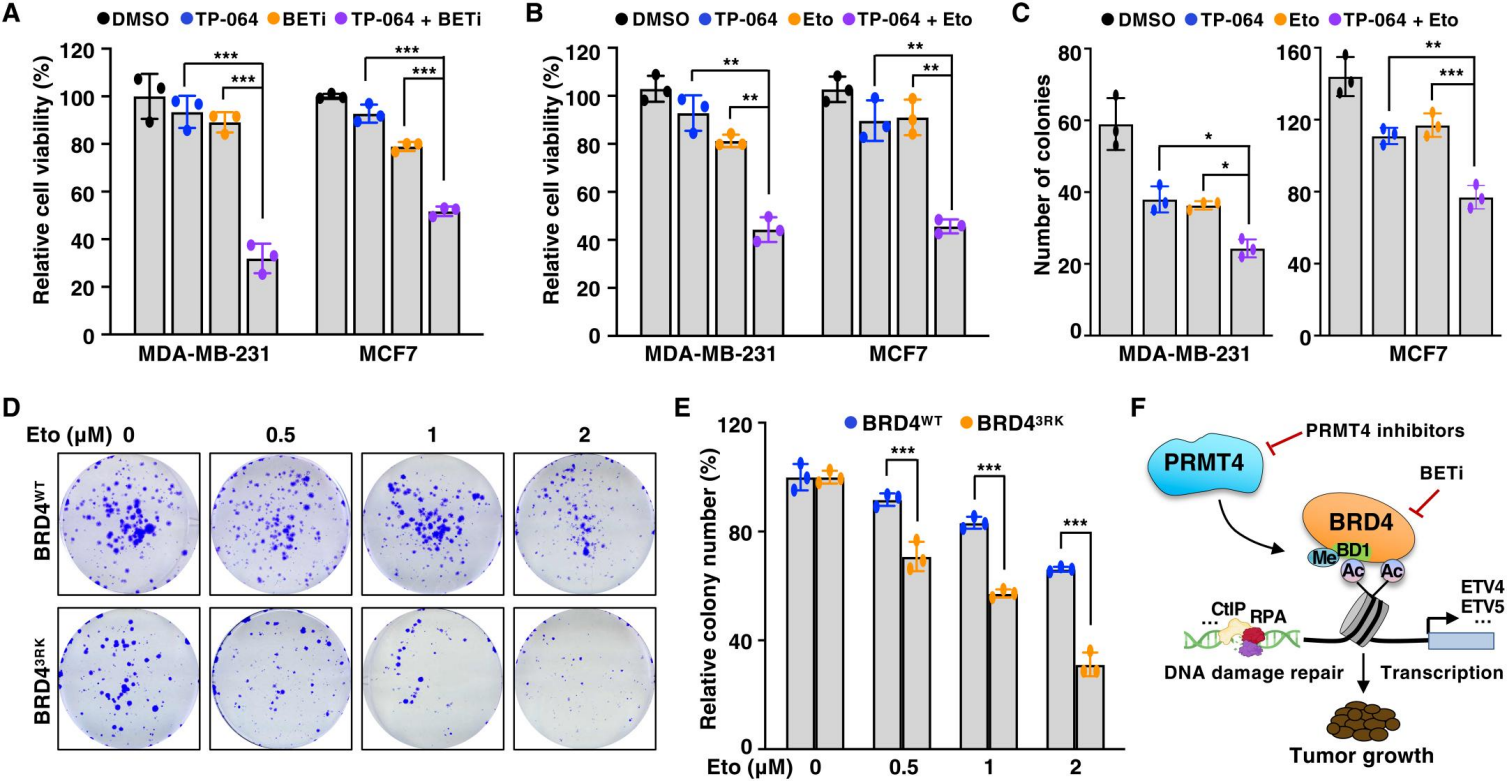
Inhibition of BRD4 arginine methylation sensitizes cells to BETi and DNA damage agent. (**A**) Cells were treated with I-BET151 (BETi) (0.25 μM for MCF7 and 0.5 μM for MDA-MB-231) and TP-064 (10 μM for MCF7 and 5 μM for MDA-MB-231) for 4 days and then subjected to cell viability assays. Data are means ± SD (*n* = 3). (**B**) MDA-MB-231 and MCF7 cells were treated with dimethyl sulfoxide (DMSO), 10 μM TP-064, 1 μM Eto, or both for 4 days and then subjected to cell viability assays. Data are means ± SD (*n* = 3). (**C**) MDA-MB-231 and MCF7 cells were treated with 10 μM TP-064 for 2 days followed by treatment with 1 μM Eto for 2 hours and then subjected to colony formation assays. Colonies were quantified. Data are means ± SD (*n* = 3). (**D** and **E**) BRD4^WT^ and BRD4^3RK^ knock-in MDA-MB-231 cells were treated with indicated doses of Eto for 2 hours and then subjected to colony formation assays. Representative images were shown in (D). Colonies were quantified in (E). Data are means ± SD (*n* = 3). (**F**) A proposed model depicting PRMT2/4-mediated methylation of BRD4 in transcription regulation, DNA damage repair, and tumor growth. In (A) to (C) and (E), *P < 0.05, **P < 0.01, and ***P < 0.001, two-tailed *t* test.

BETi was designed on the basis of its competition with acetylated lysine binding of BET proteins, thereby leading to the displacement of BET proteins from chromatin (*5*). Given that PRMT4 regulates BRD4 binding to chromatin, we tested whether PRMT4 inhibition affects BETi sensitivity in breast cancer cells. Compared to monotreatment, cotreatment of TP-064 with BETi significantly decreased cell viability (Fig. 6A). In agreement with the finding that deficiency in BRD4-3R methylation impairs DNA repair (Fig. 4), TP-064 sensitized breast cancer cells to etoposide in terms of cell viability and colony formation (Fig. 6, B and C, and fig. S8A). We also observed an additional antiproliferative effect with the combination of TP-064 and cisplatin (fig. S8, B to D). Consistently, BRD4^3RK^ cells were more sensitive to etoposide than BRD4^WT^ cells (Fig. 6, D and E), which is in part due to enhanced apoptosis (fig. S8E). These results suggest that PRMT4 inhibition enhances the efficacy of BETi and DNA damaging agents in part by blocking BRD4 arginine methylation, which might have synergistic effects with methylation blockage of other PRMT4 substrates. Therefore, targeting PRMT4 is a potential option for improvement of sensitivity to BETi and DNA damaging agents in breast cancer. Collectively, our study reveals a previously unrecognized regulatory mechanism elucidating that PRMT2/4-mediated arginine methylation of BRD4 directly promotes the BD1 binding to acetylated histones/chromatin and consequently regulates BRD4-mediated transcription, DNA repair, and tumor growth (Fig. 6F).

## DISCUSSION

A recent study revealed that inhibition of the BD1, but not the BD2, phenocopied the effects of BETi in suppressing cell proliferation and survival in cancer cell models (*56*), suggesting the essential role of the BD1 for the oncogenic function of BRD4. Although previous studies have shown that the BD1 could be phosphorylated at Y97/Y98 by JAK2 and methylated at K99 by SETD6, these PTMs do not affect the BD1 binding to acetylated histones (*57*, *58*). Another study showed that PRMT4 inhibitors decreased BRD4 genomic occupancy at the super-enhancers likely by disrupting the interaction between methylated BAF155 and BRD4 (*59*). However, this is not the sole mechanism underlying PRMT4-mediated regulation of BRD4 because less than 6% of BRD4 forms a complex with methylated BAF155 as shown in their study. Our results demonstrate that PRMT4 directly methylates BRD4 to control its binding to chromatin and enhancer/promoter, providing another layer of regulation. It has been also reported that casein kinase II–mediated phosphorylation of BRD4 at the N-terminal cluster of phosphorylation sites (NPS), which are located downstream of BD2, induced conformational changes to regulate the chromatin-binding activity of BD2 (*60*). Given that 3R are located downstream of BD1, it is possible that like NPS phosphorylation, PRMT2/4-mediated 3R methylation also causes BRD4 local conformational change and consequently provides BRD4 access to acetylated chromatin, which is warranted in the future studies.

BRD4 has been proposed as a major guardian of genome stability through both transcription-dependent and transcription-independent mechanisms. It regulates transcription of several key DNA repair genes involved in HR and nonhomologous end joining, such as Rad51, BRCA1, CtIP, XRCC4, and LIG4 (*43*, *61*, *62*). BRD4 also acts as a scaffold to recruit repair proteins to DNA damage sites, including 53BP1, KU70, and KU80 (*43*, *47*). However, it remains elusive how BRD4 function is regulated in response to DNA damage. Our finding reveals that arginine methylation of BRD4 by PRMT2/4 represents a critical regulatory mechanism that dynamically modulates BRD4 function on DDR signaling.

Down-regulation of key oncogenes that are associated with enhancer/super-enhancers is considered as the primary mechanism for the anticancer activity of BETi (*5*). We found that arginine methylation of BRD4 promotes the mRNA expression of ETV4 and ETV5 by enhancing BRD4 enrichment on the enhancer/promoter region. ETV4 and ETV5, together with ETV1, belong to the PEA3 subfamily of ETS transcription factors (*63*). They are overexpressed in numerous types of cancers and associated with tumor aggressiveness, poor prognosis, and drug resistance (*64*). Thus, ETV4 and ETV5 may act as key downstream effectors of BRD4 to promote tumor growth and dictate BETi efficacy. Moreover, despite the fact that BRD4 is a promising target for anticancer therapeutics and numerous BETi have been evaluated in clinical trials, these BETi have achieved limited success as monotherapy due to toxicities and emergence of resistance (*10*–*12*). We showed that PRMT4 inhibition increases sensitivity to BETi and DNA damaging agents in breast cancer cells. Hence, our study highlights the combination of PRMT4 inhibitors and BETi or DNA damaging agents as a potential strategy for cancer therapy.

We observed that knockdown of BRD4 increases protein levels of PRMT2/4 (Fig. 2A). Consistently, RNA-seq data showed that the mRNA levels of PRMT2 and, to a lesser extent, PRMT4, are elevated upon BRD4 depletion. However, the mRNA levels of other PRMTs were decreased in BRD4-depleted cells. Moreover, knockdown of BRD4 also led to an increase of PRMT2/4 recruitment to chromatin (Fig. 3B), which is possibly due to that BRD4 depletion increases PRMT2/4 expression, or BRD4 depletion increases free PRMT2/4 pool, or BRD4 may compete with PRMT2/4 on binding of certain chromatin region. These observations suggest a possible feedback regulation of PRMTs by BRD4, which warrants future studies.

## MATERIALS AND METHODS

### Cell culture and reagents

HEK293T, HEK293, MCF7, and MDA-MB-231 cells were obtained from American Type Culture Collection. All cells were cultured in Dulbecco’s modified Eagle’s medium containing 10% fetal bovine serum, penicillin (100 U/ml), and streptomycin (100 μg/ml) and maintained at 37°C and 5% CO_2_. Etoposide (E1383) and I-BET151 (SML0666) were purchased from Sigma-Aldrich. TP-064 (6008) was purchased from Tocris Bioscience. EZM2302 (HY-111109) was purchased from MedChemExpress.

### Transfection, lentivirus production, and infection

For protein expression, transfection was performed using Lipofectamine 3000 (Thermo Fisher Scientific, L3000001) according to the manufacturer’s instructions. For lentivirus production, target constructs containing single guide RNA (sgRNA), shRNA, or complementary DNA (cDNA) were cotransfected with packaging plasmids (pMD2G and pSPAX2) into HEK293T cells with polyethylenimine (Polysciences, 23966-1). Twenty-four hours after transfection, fresh medium was replaced. Virus containing supernatants were harvested at 48 hours after transfection and filtered with 0.45-μm polyethersulfone (PES) filter. Targeted cells were infected with virus and selected with hygromycin (200 μg/ml), puromycin (2 μg/ml), or blasticidin (10 μg/ml) for 4 days to eliminate the noninfected cells.

### Plasmids

Hemagglutinin (HA)–PRMT1, HA-PRMT2, HA-PRMT3, HA-PRMT4, HA-PRMT5, HA-PRMT6, HA-PRMT7, HA-PRMT8, HA-PRMT9, HA-PRMT2-ΔSH3, HA-PRMT4-PH, HA-PRMT4-CC, and HA-PRMT4-C-ter were generated by cloning the corresponding cDNA into pRK5-HA vector. Myc-BRD4, Myc-BRD4-S, and fragments (F1 to F5) of Myc-BRD4 were generated by cloning the corresponding cDNA into pRK5-Myc vector. pLenti-HA-BRD4 was generated by cloning the BRD4 cDNA into pLenti-HA vector. Glutathione *S*-transferase (GST)–BD1–3R was generated by cloning the BD1-3R cDNA into pGEX-6P-1 vector. Various mutations or deletion mutants were generated using the QuikChange XL Site-Directed Mutagenesis Kit (Agilent, 20518). shRNA of PRMT2 (shPRMT2) (TRCN0000035897 and TRCN0000035894), shRNA of PRMT4 (shPRMT4) (TRCN0000007167 and TRCN0000007169), and shRNA of BRD4 (shBRD4) (TRCN0000382028) were purchased from Sigma-Aldrich. sgRNAs targeting PRMT2, PRMT4, or BRD4 were designed at www.synthego.com and were cloned into lentiCRISPR v2 vector (Addgene, 52961). sgRNAs were listed in table S1. pDSB vector was generated from pCMV–green fluorescent protein (GFP; Addgene, 11153) using site-directed mutagenesis to introduce an Eco RV recognition site.

### Antibodies

All primary antibodies were diluted with 5% nonfat milk in Tris-buffered saline with 0.1% Tween 20 (TBST) buffer for Western blot. Anti-PRMT1 (2449), anti-PRMT4 (12495), anti-PRMT5 (79998), anti–Ki-67 (9027), anti–Myc-tag (2278), anti–histone H4 (2935), anti–phospho-Chk1-Ser^345^ (2348), anti-GST (2625), anti-HA (3724), anti–γ-H2AX (9718), anti-BRD4 (13440), anti–cleaved poly(ADP-ribose) polymerase (PARP; 5625), anti–p53-pS15 (9284), anti–adme-R (13522), anti–me-PABP1 (3505), and anti-CDK9 (2316) were purchased from Cell Signaling Technology. Anti-PRMT2 (720141) and anti–histone H3 (PA5-16183) were purchased from Thermo Fisher Scientific. Anti-PRMT3 (ab191562) was purchased from Abcam. Anti-PRMT6 (sc-271744), anti–cyclin T1 (sc-271348), and anti-PABP (sc-32318) were purchased from Santa Cruz Biotechnology. Anti-PRMT7 (A12159) and anti-ETV4 (a5797) were purchased from ABclonal. Anti-HA (901503) was purchased from BioLegend. ASYM24 (07-414), peroxidase-conjugated anti-mouse secondary antibody (A4416), and anti-rabbit secondary antibody (A4914) were purchased from Sigma-Aldrich. Anti–α-tubulin (66031-1-Ig), anti-PARP1 (66520-1-Ig), and anti-NSD3 (11345-1-AP) were purchased from Proteintech. *Xenopus laevis* anti-RPA antibody was developed previously (*65*). Anti-MMA and anti-ADMA were a gift from M. Bedford at MD Anderson Cancer Center. Anti-CtIP was a gift from R. Baer at Columbia University.

### Immunoblot and immunoprecipitation analyses

Cells were rinsed with ice-cold phosphate-buffered saline (PBS) and lysed in EBC buffer [50 mM tris (pH 7.5), 120 mM NaCl, and 0.5% NP-40] or Triton buffer [40 mM Hepes (pH 7.4), 150 mM NaCl, 2.5 mM MgCl_2_, 1 mM EDTA, and 1% Triton X-100] supplemented with protease inhibitor (Thermo Fisher Scientific, A32953) and phosphatase inhibitors (phosphatase inhibitor cocktail set I and II, Calbiochem). The cell lysates were centrifuged at 13,200 rpm at 4°C for 10 min. The protein concentration of lysates was determined using NanoDrop by Bio-Rad protein assay reagent. Equal amounts of whole-cell lysates were resolved by SDS–polyacrylamide gel electrophoresis (SDS-PAGE) and immunoblotted with indicated antibodies. For immunoprecipitation (IP), 2000 to 5000 μg of lysates were incubated with agarose-conjugated antibodies for 3 to 5 hours at 4°C. Immunoprecipitants were washed three times with NETN buffer [20 mM tris (pH 8.0), 150 mM NaCl, 1 mM EDTA, and 0.5% NP-40] or Triton buffer before being resolved by SDS-PAGE. Anti-HA agarose beads (A2095) were purchased from Sigma-Aldrich. Anti-Myc agarose beads (658502) were purchased from BioLegend. Anti-BRD4 agarose beads (sc-518021) were purchased from Santa Cruz Biotechnology.

### Purification of GST-tagged protein from *Escherichia coli*

Following procedures previously described in (*27*), recombinant GST-BRD4-BD1 truncated protein was purified from BL21(DE3) *Escherichia coli*. Protein expression was induced by 0.1 mM isopropyl-β-d-thiogalactopyranoside at 25°C for 16 hours. Bacteria were collected and resuspended in GST buffer [25 mM tris (pH 8.0), 5 mM dithiothreitol, and 150 mM NaCl] and sonicated. After centrifugation, the supernatant was incubated with glutathione sepharose beads (Cytiva, 17075605) for 2 hours at 4°C, followed by three times washes with GST buffer and eluted with elution buffer [10 mM l-glutathione and 50 mM tris-HCl (pH 8.0)].

### In vitro methylation assays

Five micrograms of recombinant GST-BRD4-BD1-WT and GST-BRD4-BD1-3RK truncated proteins were incubated with HA-PRMT2, HA-PRMT4, or HA-PRMT7 in the methylation buffer [50 mM tris-HCl (pH 8.5), 20 mM KCl, 10 mM MgCl_2_, 1 mM β-mercaptoethanol, and 100 mM sucrose] with 2 μl of adenosyl-l-methionine and *S*-[methyl-3H] (1 mCi/ml stock solution, PerkinElmer) at 30°C for 1 hour. The reactions were stopped by 3× SDS loading buffer. The samples were resolved by SDS-PAGE and transferred to polyvinylidene difluoride membrane, which was then sprayed with EN3HANCE (PerkinElmer) and exposed to an x-ray film.

### Mass spectrometric analysis of BRD4-3R methylation

Following procedures previously described in (*27*), HEK293T cells were transfected with Myc-BRD4. Forty-eight hours after transfection, the cells were lysed in Triton buffer, followed by immunoprecipitation. The immunoprecipitates were resolved by SDS-PAGE and visualized using GelCode blue staining reagent (Thermo Fisher Scientific, 24590). The band containing Myc-BRD4 was excised and digested with trypsin. Peptides were analyzed on an EASY nLC 1200 in-line with the Orbitrap Fusion Lumos Tribrid mass spectrometer (Thermo Fisher Scientific). Peptides were pressure-loaded at 800 bar and separated on a C18 reversed phase column [Acclaim PepMap rapid separation liquid chromatography (RSLC), 75 μm × 50 cm (C18, 2 μm, 100 Å)] (Thermo Fisher Scientific) using a gradient of 2 to 35% B in 180 min [solvent A: 0.1% formic acid (FA); solvent B: 80% acetonitrile (ACN)/0.1% FA] at a flow rate of 300 nl/min at 45°C. Mass spectra were acquired in data-dependent mode with a high resolution (60,000) Fourier transform MS survey scan followed by MS/MS of the most intense precursors with a cycle time of 3 s. The automatic gain control target value was 4.0 × 10^5^ for the survey MS1 scan. Precursors were isolated with a 1.6 mass/charge ratio window with a maximum injection time of 50 ms. MS/MS spectra were acquired using higher-energy collisional dissociation (HCD) and electron transfer dissociation (ETD) for each peptide precursor in an alternating fashion. The HCD collision energy was 35%, and ETD was performed using the calibrated charge-dependent ETD parameters. The fragment ions were detected in the Orbitrap at a resolution of 15,000. Spectra were searched against a custom database containing human BRD4 and a database of common contaminants using MaxQuant and Proteome Discoverer. The false discovery rate, determined using a reversed database strategy, was set at 1% at the peptide and modification site levels. Fully tryptic peptides with a minimum of seven residues were required including cleavage between lysine and proline. Two missed cleavages were permitted. Sites of modification were manually verified.

Because of the amino acid sequence surrounding arginine residues 179, 181, and 183, the detection of methylation of these residues was challenged by MS/MS. In our initial analyses of the Myc-BRD4 protein, trypsin was used as it is highly efficient during in-gel digestion and yields peptides with a positive charge at the C terminus facilitating detection by LC-MS/MS. Trypsin cleaves on the C-terminal side of lysine and arginine unless the residues are modified. Trypsin digestion of the unmodified or partially modified peptide: [QAK..GR(179)GR(181)GR(183)K..ETG] leads to small peptides that are not detectable by MS. Thus, the data were inconclusive regarding modification at one or all of these sites. We were able to detect one version of the peptide with a missed cleavage at K177 that revealed methylation at R179. It is likely that other sites were modified, but the peptides were too low to detect with poor ionization or fragmentation efficiency. To address the challenge observed with trypsin, we also digested the protein with GluC that cleaves on the C-terminal side of glutamic acid. Unfortunately, we recovered very little of the protein and did not detect peptides within this part of the protein.

### RNA-seq and analysis

Total RNAs were prepared from shPRMT2/4, shBRD4, or control shRNA of GFP (shGFP) MCF7 cells with a NucleoSpin RNA Plus kit (Takara, 740984.50) following the manufacturer’s instructions. Each was in triplicate. RNA-seq was performed at the Beijing Genomics Institute using the protocol as previously described (*66*). RNA integrity was assessed using an Agilent 2100 Bio analyzer (Agilent RNA 6000 Nano Kit). The poly-adenine containing mRNA molecules were purified using poly-T oligo-attached magnetic beads. Following purification, the mRNA is fragmented into small pieces using divalent cations under elevated temperature. The cleaved RNA fragments are copied into first-strand cDNA using reverse transcriptase and random primers. This is followed by second-strand cDNA synthesis using DNA polymerase I and ribonuclease H (RNase H). These cDNA fragments then have the addition of a single “A” base and subsequent ligation of the adapter. The products are then purified and enriched with PCR amplification. The PCR yield was quantified by Qubit and pooled samples together to make a single-strand DNA circle (ssDNA circle), which gave the final library. DNA nanoballs (DNBs) were generated with the ssDNA circle by rolling circle replication to enlarge the fluorescent signals at the sequencing process. The DNBs were loaded into the patterned nanoarrays, and pair-end reads of 100 bp were read through on the DNBSEQ platform.

Raw reads were filtered by software SOAPnuke to remove reads with adaptors, reads in which unknown bases (*N*) are more than 0.1%, and low-quality reads that are defined as the percentage of base which quality is lesser than 20 is greater than 40% in a read. Clean reads were mapped to the human genome with HISAT2 and Bowtie2. The gene expression level was calculated with RNA-Seq by expectation maximization (RSEM). Differentially expression genes were detected with DEseq2. Fragments per kilobase of transcript per million (FPKM) values were tabulated and normalized for log_2_ [fold change(FPKM)], removing all zero-FPKM genes from the analysis. PRMT2, PRMT4, and BRD4 were omitted in the analysis because they were altered by shRNA. For the Venn diagram and pathway analysis, genes were assessed in each shBRD4 or shPRMT2/4 group for those within the top or bottom 20% in magnitude and having an adjusted *P* value of ≤ 0.05 from the differential expression analysis. Pathway analysis was performed using Gorilla, using the ranked list of mean log_2_ [fold change(FPKM)] values from all shBRD4 and shPRMT2/4 samples. GO term gene symbols and descriptions were obtained using AmiGO.

### Immunofluorescence staining

Immunofluorescence staining was performed according to a protocol described previously (*67*). Cells were grown on glass coverslips for 24 hours before treated with 10 μM etoposide for 1 hour or TP-064 for 2 days. Cells were fixed with 3.7% formaldehyde in PBS for 15 min at room temperature and permeabilized with 0.1% Triton X-100 in PBS for 5 min. Cells were then rinsed three times with PBS, followed by incubation with 5% bovine serum albumin for 30 min at room temperature. Cells were incubated with primary antibodies overnight, washed three times with PBS containing 0.02% Tween 20 (PBST), and then incubated with Alexa 488–conjugated anti-rabbit secondary antibody or Alexa 594–conjugated anti-mouse secondary antibody for 60 min. Nuclei were stained with 4′,6-diamidino-2-phenylindole for 10 min. Coverslips were rinsed two times with PBS and mounted onto slides using VECTASHIELD Antifade Mounting Media (Vector Laboratories).

### Generation of BRD4^3RK^ knock-in cells

Two steps were applied to generate BRD43RK knock-in cells. First, the BRD4R179K/R181K knock-in cells were generated. The sgRNA (AGGCAAAAGGAAGAGGACGT) targeting BRD4 around the R179/R181 was inserted into lentiCRISPR v2 vector. The single-stranded oligodeoxynucleotide (ssODN) containing R179K/R181K mutation was used as a template. The sgRNA vector and ssODN were cotransfected into MDA-MB-231 cells. Forty-eight hours after transfection, the cells were treated with puromycin (2 μg/ml) for 2 days and then seeded into a 96-well plate at one to two cells per well. The R179K/R181K knock-in cells were identified by PCR followed by Mae II digestion and validated by DNA sequencing. Second, the R183K knock-in mutation was generated in BRD4R179K/R181K cells. The sgRNA (AGGCAAAAGGAAAAGGAAAG) targeting BRD4 around the R183 was inserted into lentiCRISPR v2 vector. The ssODN containing R183K mutation was used as a template. The sgRNA vector and ssODN were cotransfected into BRD4-R179K/R181K knock-in cells. Forty-eight hours after transfection, the cells were selected with puromycin (2 μg/ml) for 2 days and then seeded into a 96-well plate. The R183K knock-in cells were identified by PCR followed by Mnl I digestion and validated by DNA sequencing. The ssODNs were listed in table S1.

### Quantitative real time PCR

Total RNA was extracted using a NucleoSpin RNA Plus kit (Takara, 740984.50). One microgram of total RNA was used for first-strand cDNA synthesis using the iScript reverse transcription supermix (Bio-Rad, 1708841). The real-time RT-PCR assays were performed using EvaGreen qPCR Master Mix (Biotium, 31041). Glyceraldehyde-3-phosphate dehydrogenase (GAPDH) was used for normalization. PCR primers were listed in table S1.

### Chromatin immunoprecipitation quantitative real-time PCR

Cells (1 × 10^7^) in a 10-cm dish were cross-linked by adding 1 ml of 11% formaldehyde buffer [11% formaldehyde, 50 mM Hepes (pH 7.3), 100 mM NaCl, 1 mM EDTA (pH 8.0), and 0.5 mM EGTA (pH 8.0)] directly to the media (10 ml) at room temperature for 15 min and quenched by adding 0.575 ml of 2.5 M glycine (final concentration of 0.125 M glycine). Cells were rinsed twice with ice-cold PBS and harvested with a cell scraper followed by centrifugation at 1500*g* for 5 min at 4°C. Cells were resuspended in 1 ml of ice-cold lysis buffer 1 [50 mM Hepes (pH 7.3), 140 mM NaCl, 10% glycerol, 0.5% NP-40, 0.25% Triton X-100, and 1 mM EDTA (pH 8.0)] and incubated on ice for 10 min followed by centrifugation at 2000*g* for 5 min at 4°C. The pellet was resuspended in 1 ml of ice-cold lysis buffer 2 [10 mM tris-HCl (pH 8.0), 200 mM NaCl, 1 mM EDTA (pH 8.0), and 0.5 mM EGTA (pH 8.0)] and centrifuged at 2000*g* for 5 min at 4°C. The pellet was then resuspended in 0.5 ml of ice-cold lysis buffer 3 [50 mM tris-HCl (pH 7.5), 140 mM NaCl, 1 mM EDTA (pH 8.0), 1 mM EGTA (pH 8.0), 1% Triton X-100, 0.1% sodium deoxycholate, and 0.1% SDS] and sonicated with a Branson sonicator at 50% output power for 12 cycles at 20 s each with 10 s off between cycles on ice. Five micrograms of BRD4 antibody [normal rabbit immunoglobulin G (IgG) as a control] diluted in 200 μl of PBST was incubated with the magnetic Dynabeads Protein A (Thermo Fisher Scientific, 10001D) for 10 min at room temperature and then washed once with 200 μl of PBST and resuspended in 50 μl of lysis buffer 3. Sonicated lysates were mixed with 50 μl of BRD4-conjugated Dynabeads and incubated overnight at 4°C with rotation. Immunoprecipitants were washed three times with lysis buffer 3, followed by once with each of the following buffers: high salt buffer [50 mM tris-HCl (pH 7.5), 500 mM NaCl, 1 mM EDTA (pH 8.0), 1 mM EGTA (pH 8.0), 1% Triton X-100, 0.1% sodium deoxycholate, and 0.1% SDS], LiCl buffer [20 mM tris-HCl (pH 8.0), 250 mM LiCl, 1 mM EDTA, 0.5% NP-40, and 0.5% sodium deoxycholate], and Tris-EDTA (TE) buffer [10 mM tris-HCl (pH 8.0) and 1 mM EDTA]. DNA was eluted off the beads by incubation at 65°C for 30 min in 150 μl of elution buffer. Cross-links were reversed overnight at 65°C in 150 μl of elution buffer plus 6 μl of 5 M NaCl and 2 μl of RNase A (10 mg/ml). Protein was degraded by the addition of 2 μl of proteinase K (20 mg/ml) and incubated at 60°C for 60 min. DNA was purified with the PCR Clean Up kit (Omega Bio-Tek, D6492) and used for qPCR. PCR primers were listed in table S1.

### DSB reactions in *Xenopus* egg extract

pDSB (5 ng/μl) was incubated in high-speed supernatant supplemented with ATP (adenosine 5′-triphosphate) regeneration mix [ARM; 6.5 mM phosphocreatine, 0.65 mM ATP, and creatine phosphokinase (1.6 μg/ml)] and 10 μM nocodazole at 21°C for 20 min to form prereplication complexes. Next, two volumes of nucleoplasmic extract supplemented with ARM and 3.5 mM dithiothreitol was added to promote replication. Reactions were incubated at 21°C for 45 min to complete replication. To induce DSBs, reactions were supplemented with Age I (New England Biolabs, R0552), which recognizes a single Age I site present on pDSB. Where indicated, reactions were also supplemented with [α-^32^P]dATP (0.3 μCi/μl) to label nascent strands or 750 μM TP-064.

### Isolation of DNA-bound proteins by plasmid pulldown

pDSB was replicated in extract supplemented with buffer or TP-064. Age I was added after 45 min to induce DSBs (*T* = +0 min). Reaction samples were added to LacI-coupled magnetic beads (M-280 Dynabeads; Invitrogen) suspended in LacI pulldown buffer [10 mM Hepes (pH 7.7), 50 mM KCl, 2.5 mM MgCl_2_, 250 mM sucrose, BSA (0.25 mg/ml), and 0.02% Tween 20]. Samples were rotated at 4°C for 20 min, washed three times with LacI wash buffer [10 mM Hepes (pH 7.7), 50 mM KCl, 2.5 mM MgCl_2_, BSA (0.25 mg/ml), and 0.02% Tween 20)], resolved by SDS-PAGE, and immunoblotted with the indicated antibodies.

### Analysis of DNA intermediates by agarose gel electrophoresis

Agarose gel electrophoresis was performed as previously described (*49*). For 1D agarose gel electrophoresis, 1 μl of reaction samples was diluted sixfold in Replication Stop Dye [3.6% SDS, 18 mM EDTA, 90 mM tris-HCl (pH 8.0), Ficoll (90 mg/ml), and Bromophenol Blue (3.6 mg/ml)], incubated with 20 mg of proteinase K at 37°C for 60 min, and then resolved by 0.8% agarose gel electrophoresis. For 2D agarose gel electrophoresis, 4 μl of reaction samples was diluted 10-fold in Stop Solution [50 mM tris-HCl (pH 7.5), 25 mM EDTA, and 0.5% SDS], incubated with 4 mg of RNase for 30 min at 37°C, and then incubated with 30 mg of proteinase K at 37°C for 60 min. DNA intermediates were then resolved by 0.4% agarose gel electrophoresis at 1 V/cm for 14 hours. The 1D gel was then stained with ethidium bromide (0.3 μg/ml), and individual lanes were excised. For the second dimension, 1% agarose containing ethidium bromide (0.3 μg/ml) was cast around 1D slices, and the gel was run in buffer containing ethidium bromide (0.3 μg/ml) at 4 V/cm for 15 hours at 4°C. After electrophoresis, agarose gels were dried and visualized by autoradiography. Linear and HMW molecules were quantified from the gel images shown and graphed relative to the total signal in the buffer before DSB formation (0 min). Resection of linear fragments was quantified in sections that start above the linear spot and move along the length of the comet tail. Intensity at each position was graphed relative to peak intensity in the buffer.

### Cell proliferation assays

Cells were seeded into a 96-well plate at 1000 cells per well at day 0, and cell proliferation was determined using the Cell Counting Kit-8 (APExBIO, K1018) by measuring the absorbance at 450 nm with a microplate reader.

### Colony formation and clonogenic survival assays

Cells were seeded into a six-well plate at 200 cells per well and incubated for 7 to 10 days until visible colony formation. For clonogenic survival assay, cells were seeded in six-well plates at 500 cells per well for 24 hours before treated with indicated drugs. To maintain drug effect, the medium containing drugs was replaced every 3 days for 10 to 15 days until visible colony formation. Colonies were fixed with 10% ethanol and 10% acetic acid for 30 min and then stained with 0.4% crystal violent in 20% ethanol for 30 min, followed by washing with dH_2_O (distilled water) and manually counting.

### Cell viability assays

Cells were seeded in a 96-well plate at 2000 cells per well for 24 hours and treated with indicated doses of drugs for 96 hours. Cell viability was determined using the Cell Titer-Glo luminescent cell viability assay kit according to the manufacturer’s instructions (Promega, G7570).

### Immunohistochemistry staining

Following procedures previously described in (*27*), the xenograft tumors were fixed with 10% neutral buffered formalin for 24 hours and then paraffin-embedded and processed at the Histology and Immunohistochemistry Laboratory at Medical University of South Carolina (MUSC). Formalin-fixed, paraffin-embedded sections were deparaffinized using xylene and rehydrated in graded ethanol. Sections were heated in boiled citrate buffer (pH 6.0) for 15 min. The remaining steps were performed using the ImmPRESS Excel Amplified Polymer Kit (Vector Laboratories, MP-7601). Briefly, the sections were incubated with BLOXALL Blocking Solution (SP-6000) for 10 min, washed with wash buffer [10 mM sodium phosphate (pH 7.5), 0.9% saline, and 0.1% Tween 20] for 5 min, and blocked with 2.5% normal horse serum for 20 min. Sections were then incubated with anti-BRD4 antibody (1:20,000), anti–phospho-histone H2A.X antibody (1:480), or anti–Ki-67 antibody (1:800; Cell Signaling Technology, 9027) diluted in normal horse serum overnight at 4°C. Sections were then washed with PBST buffer for 5 min and incubated with goat anti-rabbit IgG (1:500) for 15 min at room temperature. After washing with PBST, sections were incubated with ImmPRESS Polymer Reagent for 30 min followed by washing with PBST. Afterward, all sections were developed using ImmPACT DAB EqV working solution until the desired stain intensity, counterstained with hematoxylin, and mounted using SHURMount Mounting Media (General Data, 682188).

### Xenograft mouse assays

BRD4^WT^ and BRD4^3RK^ MDA-MB-231 cells (2 × 10^6^) in 50 μl of PBS were mixed with Matrigel at 1:1 ratio and then subcutaneously injected into the flank of 5-week-old female nude mice (The Jackson Laboratory). Tumor size was measured every other day with an electronic caliper. The tumor volume was calculated using the following formula: *L* × *W*^2^ × 0.5, where *L* is the longest diameter and *W* is the shortest diameter. After 23 days, mice were euthanized. Tumors were isolated, weighed, and used for immunohistochemistry (IHC) staining. All mice are housed in 22°C, 50 to 60% humidity, and a 12-hour light/12-hour dark cycle. All mouse experiments were conducted under the protocol no. IACUC-2018-00604-1 approved by the MUSC Institutional Animal Care and Use Committee.

### Statistical analysis

As indicated in the figure legends, all quantitative data are presented as the means ± SD or means ± SEM of three biologically independent experiments or samples. Statistical analyses were performed using GraphPad Prism 9 and Excel. Statistical significance was determined by two-tailed Student’s *t* test or two-way analysis of variance (ANOVA). *P* < 0.05 was considered significant.

## Notes

View/request a protocol for this paper from *Bio-protocol*.
