# Supplementary material for: Arginine methylation of BRD4 by PRMT2/4 governs transcription and DNA repair
Source: Sci Adv. 2022 Dec 7;8(49):eadd8928. doi: 10.1126/sciadv.add8928 (PMC9728970; doi:10.1126/sciadv.add8928)
Supplement: Supplementary file 1 — Figs. S1 to S8 Table S1 [file sciadv.add8928_sm.pdf]

Science Advances
AAAS

Supplementary Materials for

Arginine methylation of BRD4 by PRMT2/4 governs transcription and DNA repair

Liu Liu *et al.*

Corresponding author: David T. Long, longdt@musc.edu; Wenjian Gan, ganw@musc.edu

Sci. Adv. **8**, eadd8928 (2022)
DOI: 10.1126/sciadv.add8928

This PDF file includes:

Figs. S1 to S8
Table S1

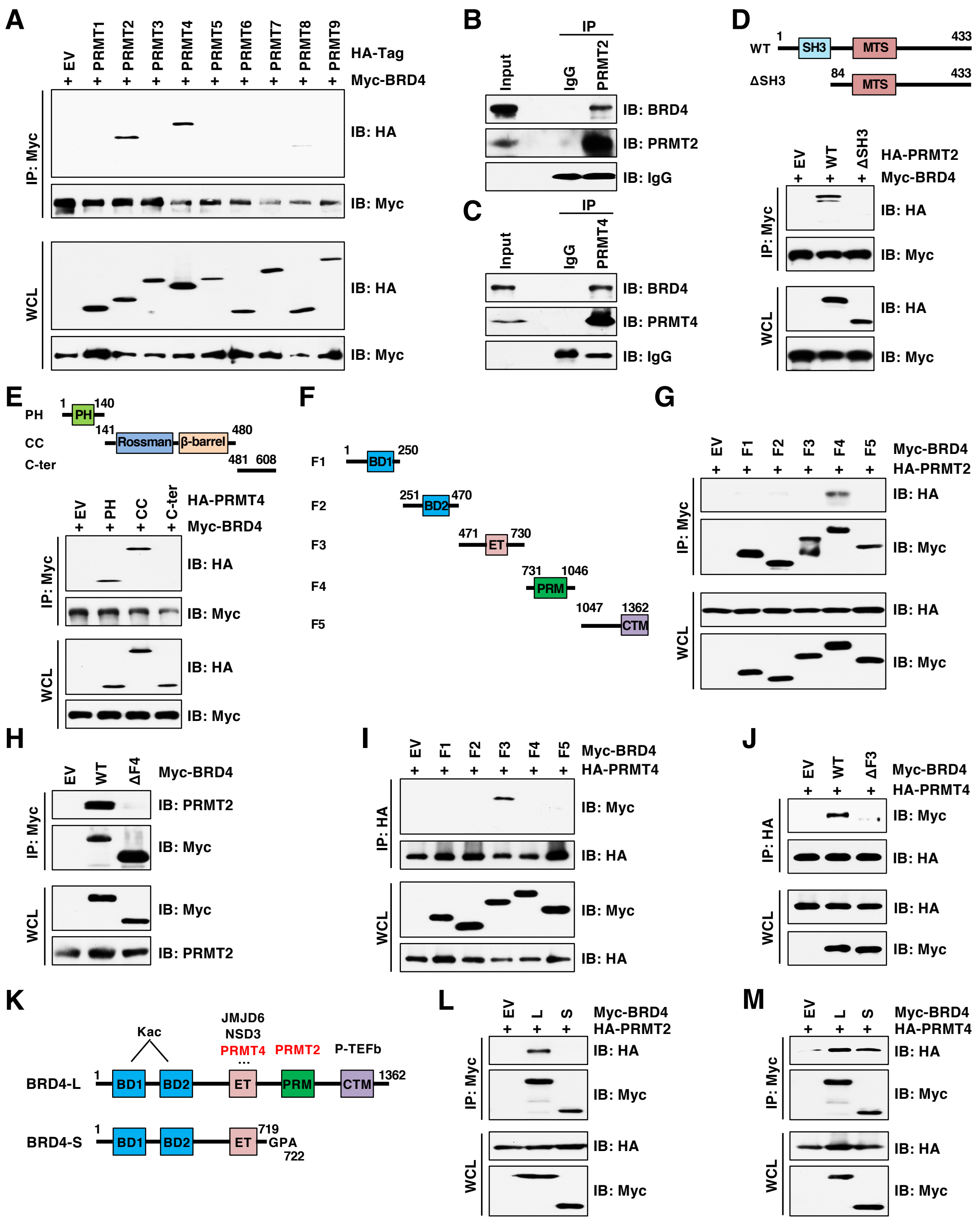

Fig. S1. BRD4 interacts with PRMT2 and PRMT4 via different domains.
(**A**) Immunoblot (IB) analysis of whole cell lysates (WCL) and Myc tag immunoprecipitation (IP) products derived from HEK293T cells transfected with HA-PRMTs and Myc-BRD4 constructs.
(**B** and **C**) IB analysis of WCL (Input) and PRMT2 (B) and PRMT4 (C) IP products derived from MCF7 cells. IgG was used as a negative control.
(**D**) A schematic presentation of PRMT2 domains (Top) and IB analysis of WCL and Myc tag IP products derived from HEK293T cells transfected with indicated constructs (Bottom).
(**E**) A schematic presentation of PRMT4 domains (Top) and IB analysis of WCL and Myc tag IP products derived from HEK293T cells transfected with indicated constructs (Bottom).

(**F**) A schematic presentation of BRD4 fragments.
(**G-J**) IB analysis of WCL and IP products derived from HEK293T cells transfected with indicated constructs.
(**K**) A schematic summary of binding partners for each BRD4 domain and BRD4-S.
(**L** and **M**) IB analysis of WCL and Myc tag IP products derived from HEK293T cells transfected with indicated constructs.

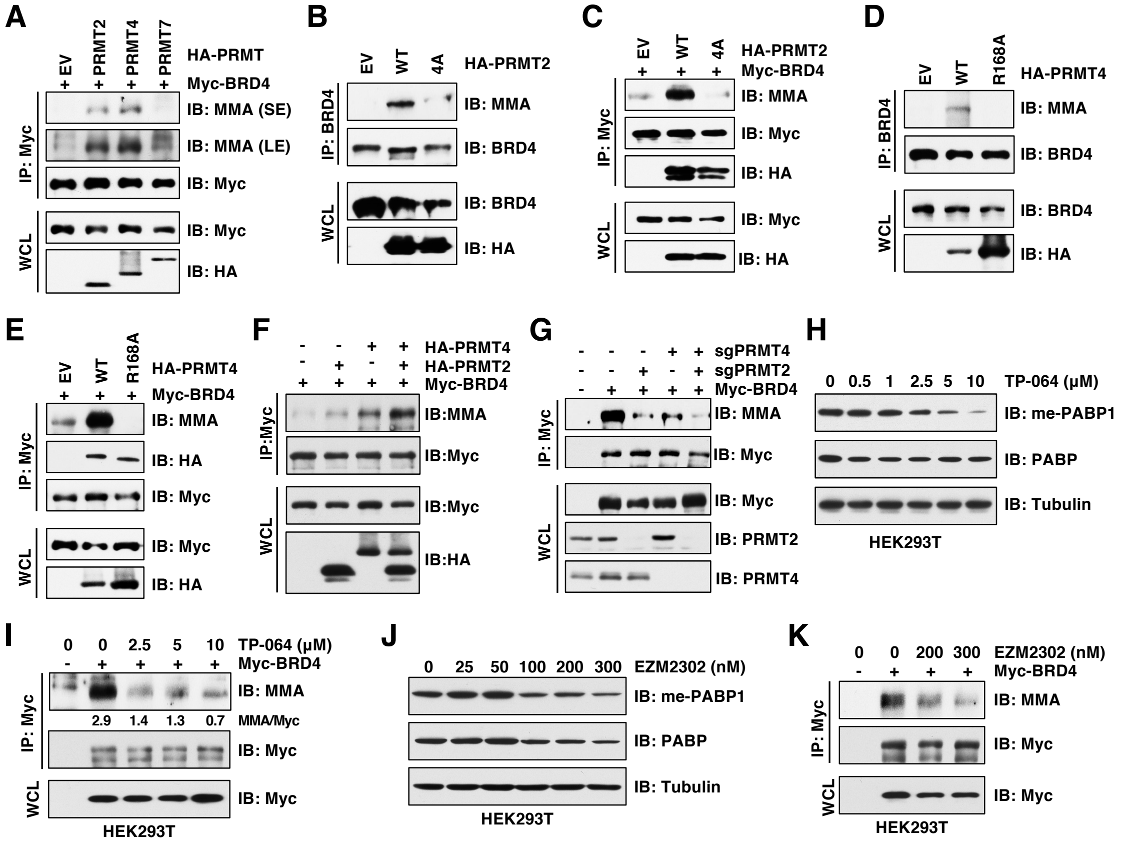

Fig. S2. PRMT2/4 catalyze BRD4 methylation.

(**A**) IB analysis of WCL and Myc tag IP products derived from HEK293T cells transfected with Myc-BRD4 and indicated HA-PRMT constructs. SE, short exposure; LE, long exposure.

(**B** and **C**) IB analysis of WCL and BRD4 IP (B) or Myc tag IP (C) products derived from HEK293T cells transfected with HA-PRMT2-WT, HA-PRMT2-4A, and/or Myc-BRD4.

(**D** and **E**) IB analysis of WCL and BRD4 IP (D) or Myc tag IP (E) products derived from HEK293T cells transfected with HA-PRMT4-WT, HA-PRMT4-R168A, and/or Myc-BRD4.

(**F**) IB analysis of WCL and Myc tag IP products derived from HEK293T cells transfected with Myc-BRD4 and HA-PRMT2, or HA-PRMT4 or both.

(**G**) IB analysis of WCL and Myc tag IP products derived from HEK293T cells transfected with Myc-BRD4. Cells were depleted of PRMT2 or PRMT4 or both before the transfection.

(**H**) IB analysis of WCL derived from HEK293T cells treated with indicated doses of TP-064 for 2 days before harvesting.

(**I**) IB analysis of WCL and Myc tag IP products derived from HEK293T cells transfected with Myc-BRD4. Cells were treated with indicated doses of TP-064 for 2 days before harvesting.

(**J**) IB analysis of WCL derived from HEK293T cells. Cells were treated with indicated doses of EZM2302 for 4 days before harvesting.

(**K**) IB analysis of WCL and Myc tag IP products derived from HEK293T cells transfected with Myc-BRD4. Cells were treated with indicated doses of EZM2302 for 4 days before harvesting.

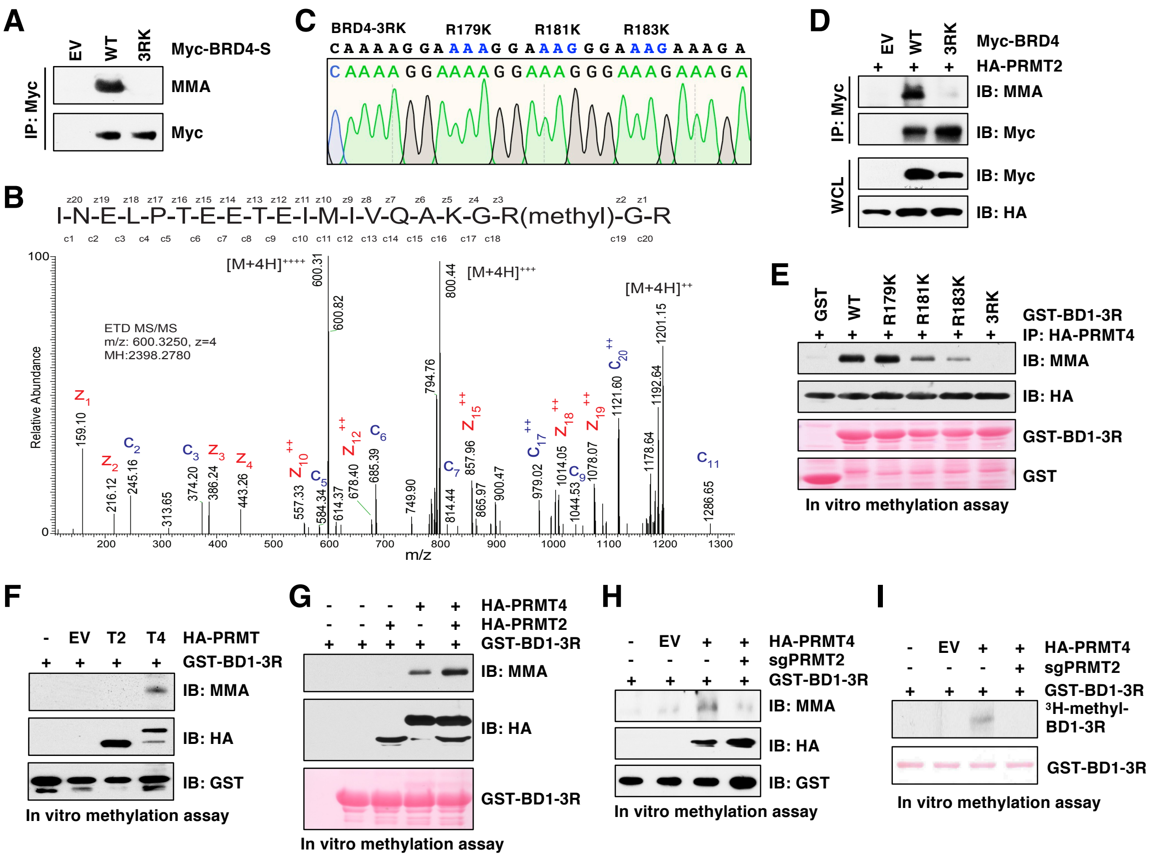

Fig. S3. BRD4 is methylated at R179, R181, and R183.

(**A**) IB analysis of Myc tag IP products derived from HEK293T cells transfected with Myc-BRD4-S-WT or 3RK constructs.

(**B**) Mass spectrometry analysis of BRD4-R179 methylation.

(**C**) Validation of the BRD4^{3RK} knock-in mutation by Sanger DNA sequencing.

(**D**) IB analysis of WCL and Myc tag IP products derived from HEK293T cells transfected with HA-PRMT2 and indicated Myc-BRD4 constructs.

(**E**) In vitro arginine methylation assays using recombinant GST-BD1-3R-WT or indicated mutants as substrates. HA-PRMT4 were immunopurified from HEK293T cells.

(**F** and **G**) In vitro arginine methylation assays using recombinant GST-BD1-3R as substrates. HA-PRMT2 or PRMT4 were immunopurified from HEK293T cells.

(**H** and **I**) In vitro arginine methylation assays using recombinant GST-BD1-3R protein as substrates. HA-PRMT4 was immunopurified from MCF7 cells stably expressing HA-PRMT4 and with or without PRMT2 depletion.

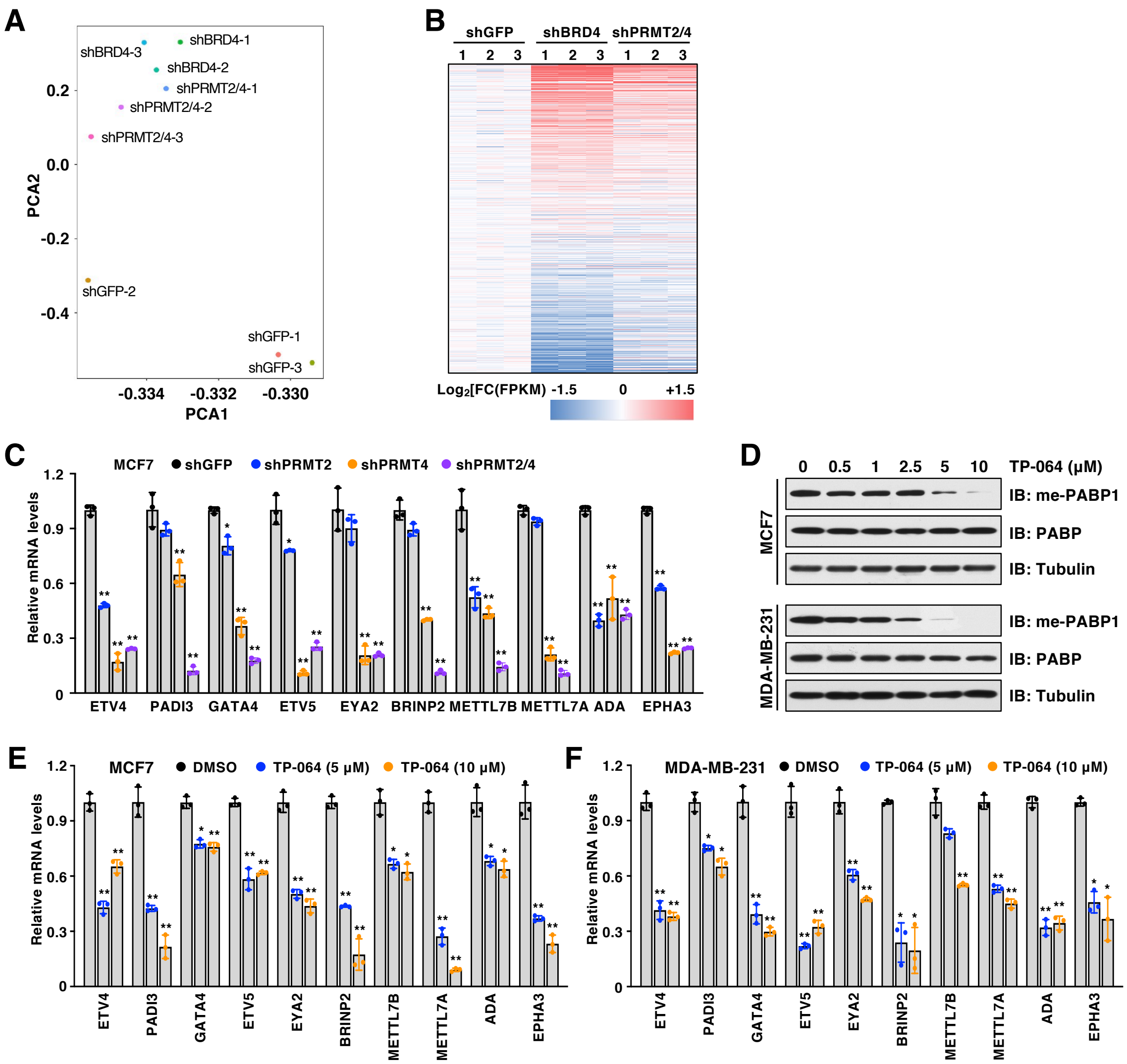

Fig. S4. RNA-seq and qRT-PCR analysis of BRD4 and PRMT2/4-dependent transcription.
(**A**) PCA analysis of gene expression in shPRMT2/4 and shBRD4 cells.
(**B**) Heatmap of differentially expressed genes in shPRMT2/4 and shBRD4 cells.
(**C**) qRT-PCR analysis of mRNA levels of indicated genes in MCF7 cells depleted of PRMT2 or PRMT4 or both. mRNA levels of these genes were normalized to GAPDH and compared to those of shGFP. Data are means ± SD (n = 3). $*p < 0.05$, $**p < 0.01$, two-tailed t test.
(**D**) IB analysis of WCL derived from MCF7 and MDA-MB-231 cells. Cells were treated with indicated doses of TP-064 for 2 days before harvesting.
(**E** and **F**) qRT-PCR analysis of mRNA levels of indicated genes in MCF7 and MDA-MB-231cells. Cells were treated with DMSO, TP-064 (5 μM or 10 μM) for 2 days. mRNA levels of these genes were normalized to GAPDH and compared to those of DMSO. Data are means ± SD (n = 3). $*p < 0.05$, $**p < 0.01$, two-tailed t test.

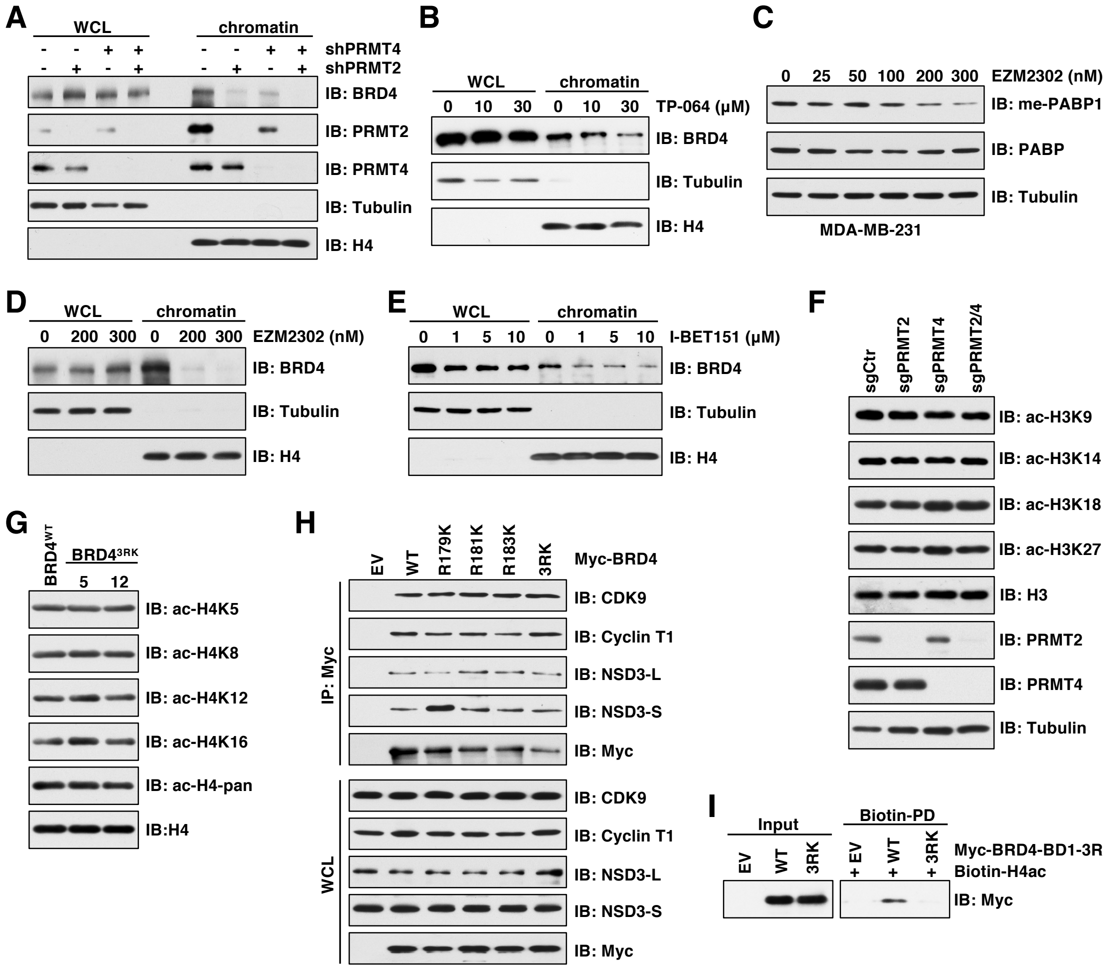

Fig. S5. BRD4 arginine methylation is required for its binding to chromatin.

(**A**) IB analysis of WCL and chromatin fraction derived from MCF7 cells depleted of PRMT2, PRMT4 or both.

(**B**) IB analysis of WCL and chromatin fraction derived from MDA-MB-231 cells treated with DMSO or TP-064 at indicated doses for 2 days.

(**C** and **D**) IB analysis of WCL chromatin fraction derived from MDA-MB-231 cells treated with indicated doses of EZM2302 for 4 days.

(**E**) IB analysis of WCL and chromatin fraction derived from MDA-MB-231 cells treated with DMSO or I-BET151 at indicated doses for 1 hour.

(**F**) IB analysis of WCL and histone extract derived from MDA-MB-231 cells depleted of PRMT2, or PRMT4, or both. sgCtr (control)

(**G**) IB analysis of histones derived from $BRD4^{WT}$ and $BRD4^{3RK}$ knock-in MDA-MB-231 cells.

(**H**) IB analysis WCL and Myc tag IP products derived from HEK293T cells transfected with indicated Myc-BRD4 constructs.

(**I**) IB analysis of pull-down products by acetylated histone H4 peptide (H4ac). The WCL derived from HEK293T cells transfected with indicated constructs was used as Input.

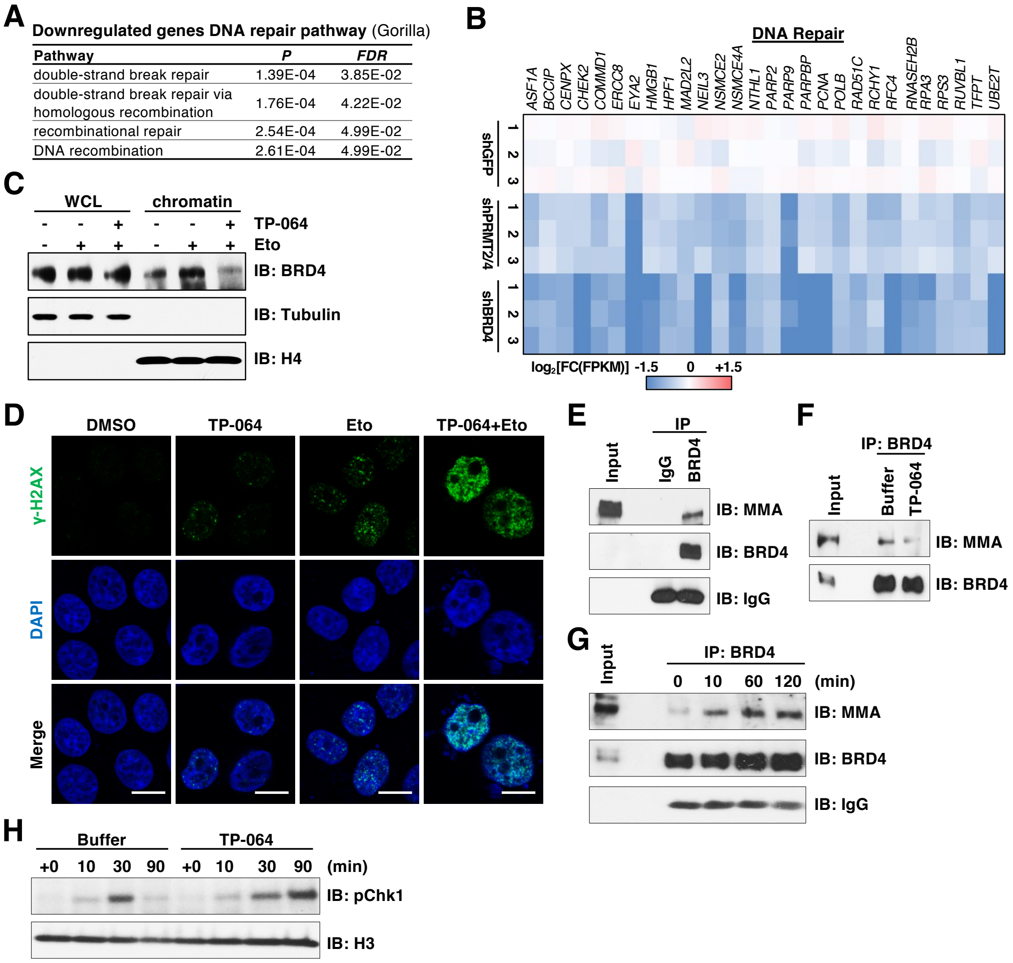

Fig. S6. PRMT4 inhibition induces DNA damage.

(**A**) Identification of DNA repair pathway downregulated by BRD4 and PRMT2/4 through GO term enrichment analysis.

(**B**) Heatmap of DNA repair genes downregulated in both shPRMT2/4 and shBRD4.

(**C**) IB analysis of WCL and chromatin derived from MDA-MB-231 cells treated with 10 μM TP-064 for 2 days and 10 μM Eto for 2 hours before harvesting.

(**D**) Immunofluorescent images of γ-H2AX in MDA-MB-231 cells treated with DMSO (control), 10 μM TP-064, 10 μM Eto or both for 60 min. Scale bar, 10 μm.

(**E**) IB analysis of input and IP products isolated from Xenopus egg extract.

(**F**) IB analysis of input and IP products isolated from extract supplemented with buffer or TP-064.

(G) IB analysis of input and IP products isolated from extract incubated with pDSB plasmid. AgeI was added to induce DSBs (T = 0 min) and samples were withdrawn at the indicated times.

(**H**) IB analysis of total samples isolated from extract. pDSB was replicated in extract supplemented with buffer or TP-064. AgeI was added to induce DSBs (T = +0 min) and samples were withdrawn at the indicated times.

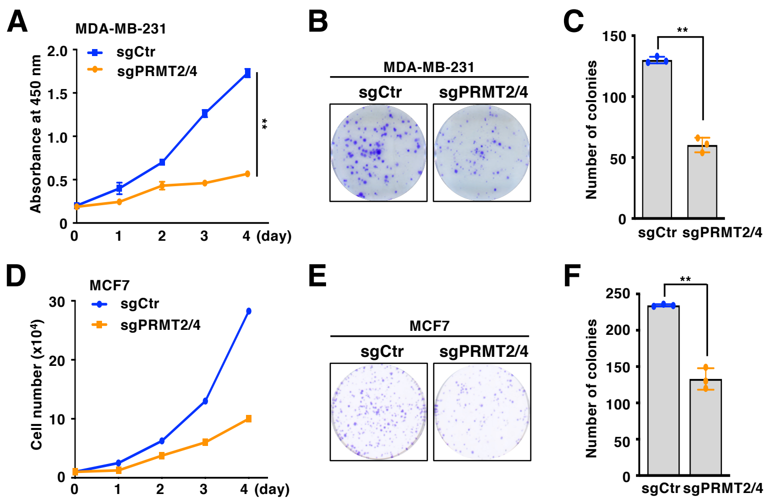

Fig. S7. PRMT2/4 depletion inhibits cell proliferation and colony formation.

(**A**) MDA-MB-231 cells depleted of PRMT2/4 (sgGFP as a negative control) were subjected to cell proliferation assays. Data are means ± SD (n = 3). **p < 0.01, Two-way ANOVA.

(**B** and **C**) MDA-MB-231 cells depleted of PRMT2/4 were subjected to colony formation assays. Representative images were shown in (B). Colonies were quantified in (C). Data are means ± SD (n = 3). **p < 0.01, two-tailed t test.

(**D**) MCF7 cells depleted of PRMT2/4 (sgGFP as a negative control) were subjected to cell proliferation assays.

(**E** and **F**) MCF7 cells depleted of PRMT2/4 were subjected to colony formation assays. Representative images were shown in (B). Colonies were quantified in (C). Data are means ± SD (n = 3). **p < 0.01, two-tailed t test.

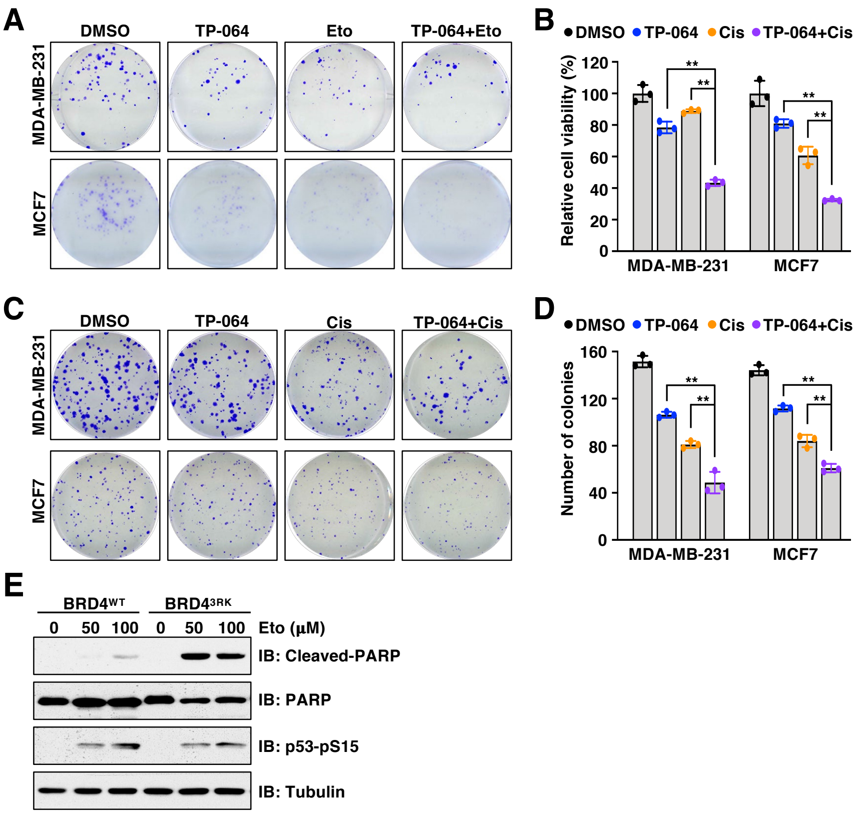

Fig. S8. Inhibition of BRD4 methylation enhances sensitivity to etoposide and cisplatin.
(**A**) Cells were treated with 10 μM TP-064 for 2 days followed by treatment of 2 μM Eto for 2 hours and then subjected to colony formation assays. DMSO as a control. Representative images were shown.
(**B**) Cells were treated with 10 μM cisplatin (Cis) and TP-064 (10 μM for MCF7 and 5 μM for MDA-MB-231) for 4 days and then subjected to cell viability assays. Data are means ± SD (n = 3). **p < 0.01, two-tailed t test.
(**C** and **D**) Cells were treated with 10 μM TP-064 for 2 days followed by treatment of 2 μM Cis for 2 hours and then subjected to colony formation assays. Representative images were shown in (C). Colonies were quantified in (D). Data are means ± SD (n = 3). **p < 0.01, two-tailed t test.
(**E**) IB analysis of WCL derived from $BRD4^{WT}$ and $BRD4^{3RK}$ cells treated with etoposide at indicated doses for 24 hours.

Table S1. Sequence of sgRNAs, ssODN and primers for quantitative real time PCR.

PRMT2-sg1	CCGGCCTCACTGCACTCAGC
PRMT2-sg2	CCTGCTGAGTGCAGTGAGGC
PRMT4-sg1	ACGGCTGCACTCTGTCTCTC
PRMT4-sg2	TGAAGGACTGCTTGCCCACA
BRD4-R179/181K-ssODN	CTACCCACAGAAGAAACCGAGATCATGATAGTCCAGG CAAAAGGAAAAGGAAAGGGGAAGAAAGAAACAGGT AGAGTACTGAAATAGCAAGTAAATTGGCTTTCT
BRD4-R183K-ssODN	CTACCCACAGAAGAAACCGAGATCATGATAGTCCAGG CAAAAGGAAAAGGAAAGGGGAAGAAAGAAACAGGT AGAGTACTGAAATAGCAAGTAAATTGGCTTTCT
ETV4-RT	Forward: 5-CAGTGCCTTTACTCCAGTGCC-3<br>Reverse: 5-CTCAGGAAATTCCGTTGCTCT-3
PADI3-RT	Forward: 5-CCTATGCGGTGCTCTACCTC-3<br>Reverse: 5-CGCTTGTCTACAAAGTTCCTGT-3
GATA4-RT	Forward: 5-GTGTCCCAGACGTTCTCAGTC-3<br>Reverse: 5-GGGAGACGCATAGCCTTGT-3
ETV5-RT	Forward: 5-CAGTCAACTTCAAGAGGCTTGG-3<br>Reverse: 5-TGCTCATGGCTACAAGACGAC-3
EYA2-RT	Forward: 5-CAGCACAAGCCTATGGAATCC-3<br>Reverse: 5-GCTGAGGAATCCACTCTGGC-3
BRINP2-RT	Forward: 5-AGTACGGCACTCATTTCTTACTT-3<br>Reverse: 5-CCTCCTGTTGTCTCTGTCTTTC-3
METTL7A-RT	Forward: 5-CAGAGTGCTGAGACCGGGA-3<br>Reverse: 5-TGGTCAGGTTGCACCCATC-3
METTL7B-RT	Forward: 5-CTCTGGTGCTGTGCTCTGTG-3<br>Reverse: 5-TGGGCTCGAAAACTTGCTG-3
ADA-RT	Forward: 5-TCACCCTTCCAGACTTCCTG-3<br>Reverse: 5-CTGTACCGCACCTCCACATAC-3
EPHA3-RT	Forward: 5-CTTCCAGGTCTCAGGACTTATG-3<br>Reverse: 5-CACCTGCTCCAACAACTTTATC-3
GAPDH	Forward: 5-GGTGTGAACCATGAGAAGTATGA-3<br>Reverse: 5-GAGTCCTTCCACGATACCAAAG-3
ETV4-ChIP-1	Forward: 5-CAAAATGCCAACCTCAGCCC-3<br>Reverse: 5-CTCACCGGGCTTCATTCCTT-3
ETV4-ChIP-2	Forward: 5-CATTTCCGGGCGATTTCTGC-3<br>Reverse: 5-AACCTCGTCCCCCAGACTAA-3
ETV4-ChIP-3	Forward: 5-CCAGGCTTTTCCTCAAGGGT-3<br>Reverse: 5-GAAGATGGCTGAGAGGGAGC-3
ETV4-ChIP-4	Forward: 5-AGGTGGCTTTGAGTTCCTCG-3<br>Reverse: 5-CACACACTCACCCTTCCCTC-3
